# Knowledge Gap Illustrations Spark Curiosity

**DOI:** 10.5334/joc.501

**Published:** 2026-05-07

**Authors:** Luisa Frede, Lisa Bardach, Younes Strittmatter, Eileen Richter, Marie Mueckstein, Markus W. H. Spitzer

**Affiliations:** 1 Department of Psychology, Martin-Luther University Halle-Wittenberg, Halle, Germany; 2 Department of Psychology, University of Giessen, Giessen, Germany; 3 Department of Psychology, Princeton University, Princeton, USA

**Keywords:** Decision making, Motivation, Learning

## Abstract

Knowledge gaps elicit curiosity and increase people’s willingness to invest resources in seeking information to close them. While previous research used confidence ratings to implicitly infer knowledge gaps, the impact of making these gaps explicitly salient to people on their information-seeking behavior remains unclear. Here, we investigated this question across two preregistered experiments (Experiment 1: *n* = 501, Experiment 2: *n* = 511 participants). Participants first took a knowledge test on elephant conservation in Botswana. Subsequently, participants repeatedly decided to either read or skip a chapter of an article covering different subtopics on elephant conservation in Botswana, before they took the same test again. We manipulated the salience of knowledge gaps by illustrating pretest performance scores to the experimental group, while the control group received no such information. In Experiment 2, we instructed participants after the pretest that a posttest would follow at the end of the experiment and thereby intended to increase the utility of information seeking. Our results provide converging evidence that illustrating moderate knowledge gaps significantly increased participants’ probability of seeking information by reading the chapters. In Experiment 2, illustrating moderate knowledge gaps was not only associated with increased information-seeking behavior, but also led to significantly greater gains in posttest performance compared to the control group. Altogether, our findings open new avenues for research on leveraging knowledge gap illustrations to deliberately stimulate curiosity, thereby increasing information-seeking behavior and knowledge acquisition.

## Introduction

Curiosity is often described as an intrinsic motivational drive to seek new information (Berlyne, 1950; Dubey & Griffiths, 2020; Grossnickle, 2016; Gruber et al., 2014; Hsiung et al., 2023; Jach et al., 2024; Kang et al., 2009; Kidd & Hayden, 2015; Kim et al., 2024; Litman, 2005; Loewenstein, 1994; Murayama, 2022; Murayama et al., 2019; van Lieshout et al., 2020; Wilson, 2024; Yagi et al., 2023). Theoretical accounts converge on the idea that curiosity arises when individuals detect a gap in their knowledge, and is satisfied once this knowledge gap is closed (Berlyne, 1950; Dubey & Griffiths, 2020; Loewenstein, 1994). These accounts are supported by empirical work showing that curiosity peaks in the presence of knowledge gaps (Brod & Breitwieser, 2019; Dubey & Griffiths, 2020; Shin & Kim, 2019; Spitzer et al., 2024, 2025; Wade & Kidd, 2019). Critically, however, prior empirical studies typically examined knowledge gaps implicitly—using confidence self-ratings—and thereby eventually increased the salience of those knowledge gaps, albeit to an unknown extent. Thus, it remains unclear whether curiosity can be elicited when knowledge gaps are made explicitly salient, compared to conditions in which no such cues are provided. Here, we investigated whether making knowledge gaps explicitly salient—by illustrating them—can effectively elicit curiosity in humans.

### Curiosity theories addressing the role of knowledge gaps

Theoretical accounts of curiosity converge that curiosity is triggered when individuals detect a gap in their knowledge. However, these accounts diverge in their predictions about the optimal magnitude of knowledge gaps for eliciting maximal curiosity. According to the information-gap theory, curiosity arises when people become aware of a discrepancy between their current and their desired knowledge (Loewenstein, 1994). This perceived gap in knowledge creates cognitive tension, motivating people to seek out new information to close the knowledge gap. Importantly, for curiosity to arise, some prior knowledge is necessary to recognize a knowledge gap. When individuals know very little about a topic, they often lack the context to identify what is missing—essentially, they don’t know what they don’t know. This results in a non-linear relationship between the magnitude of the knowledge gap and the intensity of curiosity: when individuals have no relevant knowledge (i.e., the gap is too large), curiosity is not triggered; conversely, when they already possess most of the relevant knowledge (i.e., the gap is too small), curiosity is also not triggered. Thus, the “sweet spot” for eliciting curiosity lies at a moderate knowledge gap—where enough is known to recognize what is missing, but enough is unknown to stimulate curiosity. Once knowledge gaps are closed, people’s curiosity vanishes.

Empirical evidence supporting this theory comes from Kang et al. (2009), who operationalized knowledge gaps by asking participants how confident they were in knowing the answers to questions they posed. In particular, Kang et al. (2009) prompted a set of 40 trivia questions (e.g., *“What instrument was invented to sound like a human singing?”*; answer: *Violin*) to participants and asked them to rate both their confidence in knowing the answer and their curiosity about each question (without showing the answer to participants first). In line with the information-gap theory, they observed an inverted U-shaped relationship between confidence and curiosity: when participants indicated low or high confidence in knowing the answer to the prompted question, their curiosity was relatively low. In contrast, participants’ curiosity was highest when their confidence in knowing the answer was moderate. In addition to these questions, Kang et al. (2009) also provided behavioral evidence for the link between curiosity and information-seeking behavior. They demonstrated that higher levels of curiosity increased participants’ subsequent willingness to spend resources (i.e., time or tokens) to satisfy their curiosity by finding out answers. Importantly, these findings have been replicated by several other research groups, substantiating their robustness (Brod & Breitwieser, 2019; Dubey & Griffiths, 2020; Shin & Kim, 2019; Spitzer et al., 2024, 2025; Wade & Kidd, 2019).

In contrast, the novelty theory posits that curiosity is triggered by exposure to entirely new stimuli (Berlyne, 1950, 1966, mostly reflecting large knowledge gaps^1^). According to the novelty theory, curiosity drives behavior not in order to reduce an aversive state of not knowing, but rather to explore novel information (Berlyne, 1950). This has been investigated in both animals and humans. For example, children show a preference for novel visual stimuli (Fantz, 1964) and are more likely to engage with or seek out new toys (Cantor & Cantor, 1964; Smock & Holt, 1962); for similar results in rats, see Pisula & Siegel (2005).

However, the assumption that humans should always prefer novel stimuli or experiences has been put into question, as existing empirical evidence showed that, particularly in stressful situations, people choose familiarity over novelty (Litt et al., 2011; Wansink et al., 2003). Moreover, in contexts where acquiring information is uncertain, individuals intentionally avoid acquiring information, even when doing so would be beneficial (Golman & Loewenstein, 2018; Golman et al., 2017; Hertwig & Engel, 2016). For example, even when there is no cost or effort involved, many people choose not to find out their HIV status (Hightow et al., 2003; Sweeny et al., 2010; Tao et al., 1999).

Nevertheless, the information-gap theory and novelty theory have recently been integrated into one rational account of curiosity (Dubey & Griffiths, 2020). Dubey & Griffiths (2020) propose that curiosity depends not only on the gain of information but also on its expected utility. According to their account, people are most curious about stimuli they expect to yield the greatest information gain, but only when they judge that the information could be useful in the future. Crucially, Dubey & Griffiths (2020) substantiated their predictions with empirical evidence showing that the structure of the environment determines whether curiosity followed an inverted U-shaped function of confidence (supporting the information-gap theory) or a negative relationship between curiosity and confidence (supporting the novelty theory). In particular, they found that when the probability of future encounters with a stimulus is positively correlated with prior confidence (i.e., more confidently rated stimuli are expected to appear more often), curiosity tends to peak at moderate levels of confidence—in line with the information-gap theory. This reflects the classic inverted-U pattern: the medium knowledge gap is chosen since it yields the most utility. While low-confidence gaps could also be closed, they yield less utility due to the low probability of the corresponding stimuli reappearing. In contrast, when future encounters are independent of past appraisal (i.e., when fully uncertain information is equally likely to occur and confidence is not related to the probability of occurrence), curiosity increases as confidence decreases—aligning with novelty-based theories. In such contexts, the optimal strategy is to explore the least-known stimuli, as they are expected to yield the greatest information gain.

As such, the rational account proposes that whether curiosity peaks at moderate or low confidence depends on the context and its associated utility. It thereby reconciles the information-gap and the novelty theory as special cases within a broader, utility-maximizing framework. Crucially, this account suggests that both large and moderate knowledge gaps can elicit curiosity, depending on the expected utility of the information (also see Spitzer et al., 2024).

### How can we deliberately spark curiosity?

Across theoretical accounts, curiosity is consistently linked to the presence of knowledge gaps. Prior research has estimated such gaps indirectly through confidence self-ratings, which may prompt individuals to reflect on what they do or do not know, thereby increasing the salience of their knowledge gaps—although the extent of this effect remains unclear. This raises the question: can curiosity be sparked by making knowledge gaps explicitly salient by illustrating them?

Recent work by Metcalfe et al. (2023) provides compelling support for this idea. In a series of ten experiments, participants first answered general knowledge questions and rated their confidence in each response. They then received one of several types of feedback, one of which was clear yes/no feedback indicating whether their answer was correct or incorrect. After receiving clear yes/no feedback, participants rated how curious they were to find out the correct answer. A consistent pattern emerged: when participants were highly confident but then learned they were incorrect, their curiosity to find out the correct answer increased significantly. In contrast, when they learned they were correct, curiosity vanished. These findings suggest that externally provided cues about the presence or absence of knowledge gaps strongly influence curiosity.

More traditional forms of feedback such as progress or correctness indicators provide learners with information on how much of the material they have completed or whether their answers are correct. While these feedback mechanisms can enhance engagement and reduce cognitive avoidance tendencies (Devine & Otto, 2022; Hattie & Timperley, 2007), they do not necessarily stimulate curiosity or motivation (Wisniewski et al., 2020). By contrast, feedback specifically designed to indicate gaps in knowledge may be more effective in promoting curiosity. Even though Butler & Winne (1995) did not focus on curiosity, they proposed that feedback is beneficial when it helps learners recognize discrepancies between their current understanding and the correct information, prompting them to actively work to close these gaps. This discrepancy-based mechanism aligns conceptually with the information-gap theory.

Building on this work, we theorize that the explicit salience of knowledge gaps plays a key role in sparking curiosity. Specifically, we propose that illustrating knowledge gaps to individuals increases curiosity, while illustrating that no knowledge gap exists (i.e., full knowledge has been attained) reduces it. This approach extends previous research by moving beyond confidence ratings and toward a more systematic investigation of whether curiosity can be deliberately sparked by illustrating of knowledge gaps.

### The present study

In this study, we examined whether making knowledge gaps explicitly salient elicits curiosity. In particular, we examined whether knowledge gap illustrations spark curiosity, reflected by an increased information-seeking behavior, ultimately resulting in increased knowledge gains.

Across two preregistered experiments, both of which were preceded by a pilot experiment with approximately *n* = 300 participants each (see the Online Supplement), participants first completed a pretest assessing their prior knowledge about elephant conservation in Botswana across six subtopics. Participants answered three single-choice questions about each subtopic. Next, they made a series of decisions about whether to read or skip individual chapters on these subtopics about elephant conservation in Botswana. Each chapter contained the full information to answer all questions about each subtopic. Finally, participants completed a posttest identical to the pretest.

**Figure 1:**
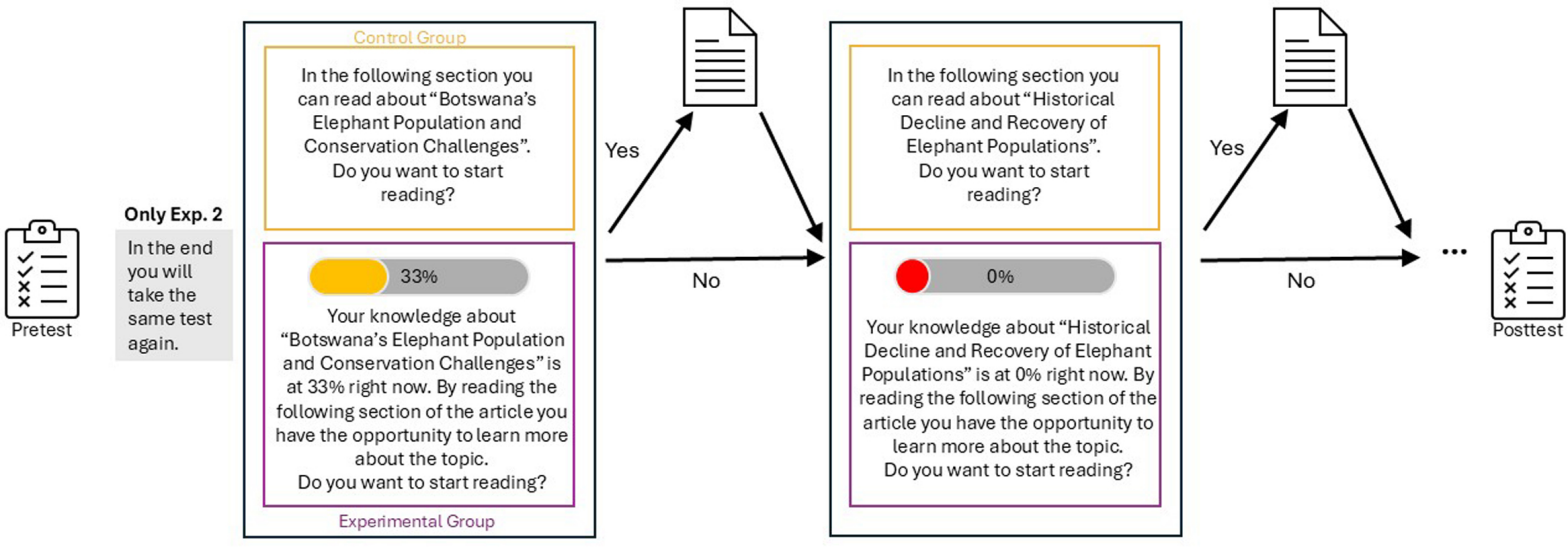
A process diagram of the experimental paradigm for the experimental group and control group, respectively. We illustrated knowledge gaps as bars and as a message. We presented a total of four different knowledge bars to participants (i.e., 0%, 33%, 67%, or 100%; only two possibilities presented here).

Participants were randomly assigned to one of two groups. In the experimental group, participants received a knowledge gap illustration before each decision—this cue indicated their current knowledge level based on pretest performance for each chapter (i.e,. 0%, 33%, 67%, or 100% prior knowledge). In the control group, we only presented the title of the next chapter and the choice to read or skip were presented, without any knowledge gap illustration (see Figure 1).

This experimental design allowed us to assess the effect of knowledge gap illustrations on participants’ probability to read (vs. skip) a chapter (i.e., information-seeking behavior). We further evaluated whether an increased information-seeking behavior was accompanied by increased knowledge gains (i.e., the difference between pretest and posttest scores).

Based on the information-gap theory, we predicted that illustrating moderate knowledge gaps (33% or 67% prior knowledge) would increase the probability to read chapters compared to no illustration. In contrast, illustrating full knowledge (100% prior knowledge) should reduce curiosity, resulting in a lower reading probability than in the control group. This should result in an inverted U-shaped relationship between prior knowledge and the probability to read in the experimental group but rather no relationship between these two variables in the control group.

According to the rational account of curiosity, the expected utility of acquiring information moderates curiosity for large knowledge gaps. Thus, our predictions for 0% prior knowledge differed between Experiment 1 and Experiment 2. In Experiment 1, participants were not told that a posttest would follow, implying relatively low utility for gaining information. Here, we hypothesized that illustrating large knowledge gaps would have no effect on participants decision to read chapters. In Experiment 2, we increased the perceived utility of gaining information by explicitly instructing participants that a posttest would follow. In this context, we hypothesized that illustrating large knowledge gaps would significantly increase the decision to read in the experimental group compared to the control group.

Additionally, we expected that the decision to read chapters should generally lead to knowledge gains across groups (except for the 100% prior knowledge condition, where improvement was not possible).

Finally, we predicted that the experimental group should gain significantly more knowledge than the control group in conditions where they also read significantly more than the control group.

## Experiment 1

The aim of Experiment 1 was to test the hypothesis that illustrating moderate knowledge gaps (i.e., 33% and 67% prior knowledge) sparks curiosity, reflected by an increased information-seeking behavior. We therefore expected an inverted U-shaped relationship between prior knowledge and the probability to read in the experimental group but not the control group. We further asked whether such amplified information-seeking behavior was accompanied with increased knowledge gains.

We preregistered Experiment 1 (AsPredicted #218945, preregistered on 03/22/2025, 04:46 AM [PT]) and intended to collect a final sample size of 200 participants (after applying the exclusion criteria). Critically, we preregistered to only include participants showing variability in their reading behavior (i.e., to skip and read at least once; but see the Online Supplementary for the results of the full data set which replicated the major findings of this experiment). The sample size was estimated based on a power analysis based on the pilot experiment. In particular, we fitted the model on the probability to read between groups using the simr package (Green & MacLeod, 2016). We ran 100 simulations and targeted an alpha level of *α* = .05. The power simulation was based on the quadratic term of the interaction effect of prior knowledge and group on the *probability to read* from the pilot experiment (an estimate of *β* = 10.79, see the Online Supplementary), as this interaction indicated an overall differential relationship between prior knowledge and the probability to read between the two groups. This power analysis indicated a power of 99% with a 95% confidence interval (CI) between 94.6% and 100%. We decided to collect a rather conservative sample size of *n* = 500 participants for Experiment 1, as we expected to exclude about half of the participants (similar to the pilot experiment). As preregistered, we excluded participants who either always chose to read or always chose to skip throughout the experiment.

### Method

#### Participants

We collected data from *n* = 501 participants (age range = 18–45; mean age = 24.3; 250 females; 251 males) online via Prolific. Participants received US$2 for participating in the experiment. The experiment lasted for 11.9 minutes on average (*SD* = 8.0). All participants participated voluntarily and gave consent that their anonymous data could be stored and published before the start of the study. Participants were told that they could stop the experiment at any time without providing a reason. Experiment 1 as well as all other experiments were conducted in English.

#### Stimuli

Experiment 1 consisted of three parts: a pretest, a decision and reading phase, and a posttest. The pretest and posttest each contained a total of 18 single-choice questions, with three questions per chapter and four possible answers per question (for the full article and the 18 questions, see the Online Supplement). The questions were identical across the two tests. The questions covered key elements from the reading material that participants could read between the two tests, and assessed participants’ knowledge on the respective chapters. The reading task consisted of an article comprising six chapters that were similar in length, each of which covered a different subtopic about elephant conservation in Botswana.

### Procedure

Figure 1 illustrates the process participants followed during this study. After providing consent to participate in the study, participants took the pretest assessing their baseline knowledge of each of the article’s six chapters. Next, we provided participants with the choice to either read or skip each of six chapters. If participants chose to read, we provided them with the chapter and they could voluntarily decide when to continue the experiment (i.e., self-paced their reading time). If they chose to skip, we presented the next choice to read or skip a different chapter. After participants made their six decisions, they completed the posttest. We randomized the order in which chapters were presented to participants.

Crucially, we randomly assigned participants to one of two groups. We collected the same number of participants for each group^2^. In the *experimental group*, we presented participants a knowledge bar and a message indicating their current knowledge level (i.e., 0%, 33%, 67%, or 100%) on the specific chapter based on their answers in the pretest (the *knowledge gap illustration*; see Figure 1). We estimated their current knowledge based on their pretest score. However, we did not directly tell participants how we specifically estimated their knowledge. The instructions were: “Your knowledge on [*topic of the following chapter*] is at [0%, 33%, 67%, or 100%]. You now have the opportunity to learn more about the topic. Do you want to read this section or skip it?” This was designed to illustrate participants’ knowledge gaps. In the *control group*, we neither provided participants with a knowledge bar nor a message on their present knowledge. Instead, we presented the following message to the participants after each chapter: “You now have the opportunity to read more on [*topic of the following chapter*]. Do you want to read this section or skip it?” (see the Online Supplementary for all instructions).

### Dependent Variables

We considered *probability to read* as the dependent variable reflecting participants’ binary choice to read or skip each chapter (coded as 1 = *read* and 0 = *skip*).

We measured knowledge gains for each prior knowledge condition (0%, 33%, 67%, or 100%). For this, we considered the *score difference* between posttest and pretest. Values ranged from 0 to 3 points (three questions and we counted one point for each correct answer), since participants could reach 0, 1, 2 or 3 points in the pretest as well as the posttest for each chapter.

### Independent Variables

We considered participants’ *prior knowledge* as our first independent variable. Prior knowledge was assessed per chapter. Participants could score between 0 and 3 points per chapter (as we asked three questions per chapter) leading to the following four prior knowledge conditions: 0%, 33%, 67%, and 100% prior knowledge. We refer to prior knowledge conditions as large knowledge gaps (0%), moderate knowledge gaps with lower knowledge (33%), moderate knowledge gaps with higher knowledge (67%) and no knowledge gaps/full prior knowledge (100%).

We considered a dichotomous *group* variable as our second independent variable which indicated whether participants were in the experimental or in the control group (we coded the contrasts with +1 for the experimental group and -1 in the control group).

To examine whether participants’ decision to read affected their knowledge gains, we considered a third independent variable: the *decision to read*, a dichotomous variable indicating whether participants chose to read (contrast coded as +1) or skip (contrast coded as -1) a chapter. All variables were group mean-centered.

### Data Analysis

We conducted the statistical analyses using R (R Core Team, 2021). We applied the tidyverse package (Wickham et al., 2019) and the sjPlot package (Lüdecke, 2024) to visualize the results and the patchwork package (Pedersen, 2024) to group figures. Additionally, we used the lmerTest package (Kuznetsova et al., 2017) to fit hierarchical linear regression models and the lme4 package (Bates et al., 2015) to fit hierarchical logistic regression models. To examine contrasts between groups and within groups for prior knowledge conditions, we utilized the emmeans package (Lenth, 2024).

To estimate the probability to read as a function of prior knowledge and group, we ran a hierarchical logistic regression with participants’ probability to read as the dependent variable. We additionally added a second-order polynomial term for prior knowledge, for allowing a non-linear relationship between prior knowledge and probability to read. We included a random intercept for participants to account for variability in participants’ overall willingness to read chapters. In particular, we applied the following regression model:


\[
{\rm probability}\ {\rm to}\ {\rm read}\sim {\rm prior}\ {\rm knowledge}^{2} \times {\rm group}+(1\vert {\rm participant}).
\]

We expected an inverted U-shaped relationship between prior knowledge and participants’ decision to read or skip the chapter in the experimental group but not the control group. This should be reflected in a significant interaction between prior knowledge and group, especially for the second polynomial term.

We further evaluated pairwise comparisons to assess differences between the two groups for each prior knowledge condition (0%, 33%, 67%, or 100%). We accounted for multiple comparisons using the Tukey adjustment for family-wise error rate. We expected significant differences between the two groups at moderate knowledge gaps (33% prior knowledge and 67% prior knowledge), with a higher probability to read in the experimental group. We further expected a significantly lower probability to read in the experimental group compared to the control group at full prior knowledge (100%) and no differences between groups at large knowledge gaps (0%). We expected no differences between these two variables in the control group.

We substantiated these pairwise comparisons by examining, within each group, the differences in the probability to read across prior knowledge conditions. We ran these pairwise comparisons to more closely examine the shape of each curve for each group. As we expected an inverted U-shaped relationship between prior knowledge and the probability to read in the experimental group, we expected a significant difference between 0% and 33% as well as 33% and 100% prior knowledge and 0% and 67% as well as 67% and 100%. We expected no difference between 33% and 67% as these conditions both represent moderate knowledge gaps.

Next, we investigated whether the decision to read led to knowledge gains. Therefore, we first evaluated whether the decision to read generally led to significantly higher knowledge gains across groups. In particular, we fitted a hierarchical linear regression with score difference as the dependent variable, prior knowledge and the decision to read (read vs. skip) as fixed effects, and a random intercept for participants. We applied the following regression model:


\[
{\rm score}\ {\rm difference}\sim{\rm prior}\ {\rm knowledge}\times{\rm decision}+(1\vert{\rm participant}).
\]

We quantified knowledge gain differences for each prior knowledge condition by running pairwise comparisons between the two groups for each prior knowledge condition. We expected significantly higher knowledge gains when participants decided to read compared to when they decided to skip a chapter. However, we only expected this for prior knowledge conditions where participants had knowledge gaps (i.e., 0%, 33%, and 67%; not 100%), since knowledge gains are not possible when having full knowledge.

To assess knowledge gains between groups, we calculated score differences and modeled them using a hierarchical linear regression with prior knowledge and group, including their interaction as fixed effects, and a random intercept for participant. We applied the following regression model:


\[
{\rm score}\ {\rm difference}\sim{\rm prior}\ {\rm knowledge}\times{\rm group}+(1\vert{\rm participant}).
\]

Again, we ran pairwise comparisons between the two groups per prior knowledge condition. We expected the experimental group to improve significantly more from pretest to posttest, especially in those cases where the probability to read had been significantly higher.

As an additional analysis, we investigated whether illustrating knowledge gaps also influenced participants’ reading time. We conducted this analysis with a hierarchical logistic regression with reading time as the dependent variable and prior knowledge, group, and their interaction as predictors, and a random intercept for participants. We included a second-order polynomial term to allow for the suspected non-linear relationship between reading time and prior knowledge. Therefore, we applied the following model:


\[
{\rm reading}\ {\rm time}\sim{\rm prior}\ {\rm knowledge}^{2}\times{\rm group}+(1\vert{\rm participant}).
\]

We also calculated pairwise comparisons using model-based estimated marginal means to compare reading times between groups at each prior knowledge condition. The readers can find the results to this analysis in the Supplementary Material.

Finally, we ran several non-preregistered models to examine whether alternative models could better explain participants’ decision to read. To examine whether previously illustrated knowledge gaps or just the very first presented knowledge gap influenced participants’ decision to read, we ran six alternative models and compared the goodness of fit of these models with the preregistered model presented above. We compared model fits using the Bayesian Information Criterion (BIC). Differences larger than 10 in BIC values between models indicate a better fit of the model with the lower BIC (Burnham & Anderson, 2002; Neath & Cavanaugh, 2012). Table 1 lists all considered models, including their BIC. In particular, we compared the preregistered model reported in our main analysis, i.e. the effect of current-chapter prior knowledge (prior knowledge_*n*_) on the decision to read (Model 1), against an alternative model examining the effect of cumulative mean prior knowledge (prior knowledge_≤*n*_) on the decision to read (Model 2). We conducted this model comparison to examine whether previous prior knowledge better explained the decision to read rather than just current-chapter prior knowledge. Next, we tested the effect of first-chapter prior knowledge (prior knowledge_*first*_) on the decision to read (Model 3) to investigate whether the very first decision to read influenced all following decisions more than the present knowledge gap. Further, we modeled the effect of overall mean prior knowledge (prior knowledge_*n̄*_) on the decision to read (Model 4) and the effects of both prior knowledge_*n*_ and prior knowledge_*n̄*_ on the decision to read (Model 5) to examine the effects of participants’ average prior knowledge and their current prior knowledge and whether these models fit the data better than the preregistered model. Lastly, we conducted a median split (mean-centered) separating participants with low prior knowledge and high prior knowledge (prior knowledge_*split*_). We tested the effect of prior knowledge_*split*_ on the decision to read (Model 6) and the effects of both prior knowledge_*n*_ and prior knowledge_*split*_ on the decision to read (Model 7) to examine whether both factors influence participants’ decision to read. All models also contained the effect of group, interaction effects between independent variables and a random intercept for participants. Additionally, we specified all the above-mentioned models again, but included random slopes for prior knowledge. Table 1 lists these models as Model 8 through Model 14. We added random slopes in order to account for within-participant variation in prior knowledge across chapters. However, model comparisons indicated a better fit to our data without the inclusion of random slopes.“

**Table 1:** Models predicting the probability to read and associated BIC scores of each model for all experiments.


MODEL	FORMULA	EXP. 1	EXP. 2	PILOT EXP. 1	PILOT EXP. 2

Model 1	\[PK_{n}\times G+(1\mid P)\]	**1828.7**	**1674.6**	**820.8**	**763.0**

Model 2	\[PK_{\leq n}\times G+(1\mid P)\]	1966.9	1864.4	857.3	–

Model 3	\[PK_{first}\times G+(1\mid P)\]	1981.8	1923.4	–	–

Model 4	\[PK_{{\bar{n}}}\times G+(1\mid P)\]	1991.3	1919.7	–	–

Model 5	\[PK_{split}\times G+(1\mid P)\]	1981.8	1919.2	–	–

Model 6	\[PK_{n}\times PK_{{\bar{n}}}\times G+(1\mid P)\]	–	–	–	–

Model 7	\[PK_{split}\times PK_{n}\times G+(1\mid P)\]	–	–	–	–

Model 8	\[PK_{n}\times G+(PK\mid P)\]	1841.3	1685.5	832.7	774.3

Model 9	\[PK_{\leq n}\times G+(PK\mid P)\]	–	1825.8	–	–

Model 10	\[PK_{first}\times G+(PK\mid P)\]	1955.4	1855.1	–	–

Model 11	\[PK_{{\bar{n}}}\times G+(PK\mid P)\]	1970.3	1864.1	870.8	–

Model 12	\[PK_{split}\times G+(PK\mid P)\]	1959.6	1857.1	857.9	845.7

Model 13	\[PK_{n}\times PK_{{\bar{n}}}\times G+(PK\mid P)\]	–	–	–	–

Model 14	\[PK_{split}\times PK_{n}\times G+(PK\mid P)\]	–	–	–	–


*Note*. Lower BIC scores indicate better model fits. The best-fitting models with respect to BIC are marked in bold font. Models marked with “–” in a given experiment either failed to converge or resulted in a singular fit; their BIC can therefore not be reported. Independent variables are *Group* (*G*), *Current-Chapter Prior Knowledge* (*PK_n_*), *Cumulative Mean Prior Knowledge* (*PK_≤n_*), *First Chapter Prior Knowledge* (*PK_first_*), *Overall Mean Prior Knowledge* ( \[PK_{{\bar{n}}}\] ), *Prior Knowledge Group* (*PK_split_*), i.e. participants grouped into low prior knowledge and high prior knowledge by performing a median split). Models 1 through 7 contain a random intercept for participants (1|*P*). Models 8 through 14 additionally contain random slopes for Prior Knowledge per participant (*PK*|*P*).

#### Additional Analyses

We further examined whether there were potential carry-over effects from previous decisions that influenced the decision to read in the current chapter. In particular, we calculated the effects of prior knowledge and group on the probability to read and additionally included a lagged predictor indicating whether the immediately preceding chapter was read. Since this model made the exclusion of the first trial for all participants necessary (since the very first decision had no preceding decision), the number of trials that this model used was not the same as in all other models. We did not compare the BIC of this model with the other models due to the different sample sizes. We report the results in the Supplementary Material.

In addition, we also examined the effects of chapter order on the probability to read. To do this, we ran a model using a median split for chapters. Results for this model are reported in the Supplementary Material.

#### Transparency and Openness

We documented all aspects of the study in accordance with transparency standards, including criteria for data exclusion, sample size determinants (i.e., power analyses), and all experimental manipulations. The complete analysis code and dataset is located at osf.io/bk9c3. Experiment 1 was preregistered via AsPredicted (AsPredicted #218945, preregistered on 03/22/2025, 04:46 AM [PT]).

### Results

**Figure 2:**
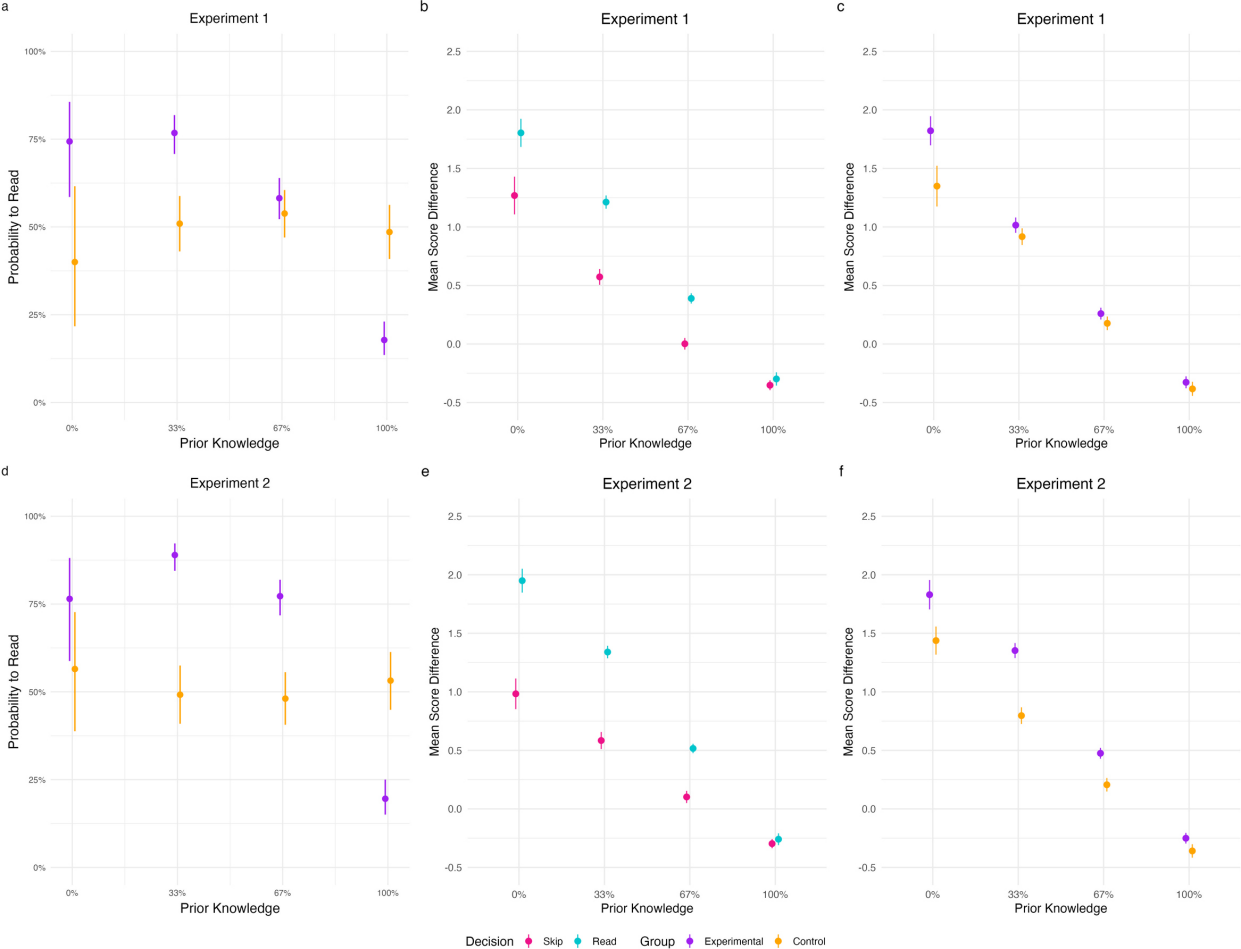
Plots a and d display the probability to read by prior knowledge and group. Plots b and e display the mean score difference by prior knowledge and decision to read. Plots c and f display the mean score difference by prior knowledge and group. Please note that mean score difference refers to the point difference between pretest and posttest scores per chapter. For each chapter, participants could achieve a score between 0 and 3 points.

The results of Experiment 1 are illustrated in Figure 2a, 2b and 2c. BIC comparisons between all models revealed that our preregistered model explained our data best in each experiment (see Table 1. Table 2 lists the distributions of participants, trials, and average trials per participant for each prior knowledge conditions and group. Note that relatively few participants fell into the 0% prior knowledge conditions. The results of the pairwise comparisons are listed in Table 3 (pairwise contrasts in the probability to read between groups), Table 4 (pairwise contrasts within groups for each pair of prior knowledge conditions), Table 5 (pairwise contrasts for knowledge gains by decision to read), and Table 6 (pairwise contrasts for knowledge gains between groups).

**Table 2:** Number of Participants, Trials, and Average Trials per Participant by Group and Prior Knowledge.


GROUP	PRIOR KNOWLEDGE	EXPERIMENTAL			CONTROL		
	
		*n*(P)	*n*(T)	*M*(T/P)	*n*(P)	*n*(T)	*M*(T/P)

**Experiment 1**	0%	30	33	1.10	14	17	1.21

	33%	92	150	1.63	74	129	1.74

	67%	125	313	2.50	93	237	2.55

	100%	120	314	2.62	91	217	2.38

**Experiment 2**	0%	22	29	1.32	23	34	1.48

	33%	82	130	1.59	61	112	1.84

	67%	129	315	2.44	81	196	2.42

	100%	126	342	2.71	80	216	2.70


*Note. n*(P) = number of participants; *n*(T) = number of trials; *M*(T/P) = average trials per participant. Values are reported separately for each group (Experimental, Control) across prior knowledge conditions (0%, 33%, 67%, 100%).

**Table 3:** Pairwise Contrasts for the Probability to Read Between Groups.


PRIOR KNOWLEDGE	EXPERIMENT 1				EXPERIMENT 2			
	
	OR	SE	*z*	*p*	OR	SE	*z*	*p*

0%	4.35	2.50	2.55	.011*	2.50	1.38	1.66	.097

33%	3.18	0.71	5.16	<.001***	8.33	2.17	8.13	<.001***

67%	1.19	0.22	0.95	.341	3.67	0.78	6.08	<.001***

100%	0.23	0.05	–6.46	<.001***	0.21	0.05	–6.60	<.001***


*Note*. Odds Ratios (*OR*) reflect the contrast: Experimental / Control for each prior knowledge condition. Values are based on estimated marginal means from hierarchical logistic regression models. *z*-values and *p*-values correspond to model-based comparisons. Significance levels: *p<.05, **p<.01, ***p<.001.

**Table 4:** Pairwise Contrasts Between Prior Knowledge Conditions Within Groups.


GROUP	CONTRAST	EXPERIMENT 1				EXPERIMENT 2			
	
		OR	SE	*z*	*p*	OR	SE	*z*	*p*

**Experimental**	0% vs 33%	0.88	0.26	–0.44	.971	0.40	0.13	–2.79	.027*

	0% vs 67%	2.08	0.85	1.80	.272	0.96	0.43	–0.10	.999

	0% vs 100%	13.38	5.28	6.57	<.001***	13.37	6.02	5.76	<.001***

	33% vs 67%	2.37	0.31	6.57	<.001***	2.37	0.36	5.76	<.001***

	33% vs 100%	15.26	3.79	10.98	<.001***	33.13	9.26	12.53	<.001***

	67% vs 100%	6.43	1.21	9.85	<.001***	13.96	2.87	12.82	<.001***

**Control**	0% vs 33%	0.64	0.24	–1.20	.629	1.34	0.42	0.95	.778

	0% vs 67%	0.57	0.29	–1.10	.688	1.40	0.59	0.81	.850

	0% vs 100%	0.71	0.32	–0.77	.869	1.14	0.44	0.35	.986

	33% vs 67%	0.89	0.13	–0.77	.869	1.05	0.13	0.35	.986

	33% vs 100%	1.10	0.25	0.43	.974	0.85	0.20	–0.68	.907

	67% vs 100%	1.24	0.23	1.15	.660	0.81	0.16	–1.07	.708


*Note*. Odds Ratios (*OR*) reflect the contrasts between prior knowledge conditions within each group (Experimental/ Control). Values are based on estimated marginal means from hierarchical logistic regression models. *z*-values and *p*-values correspond to model-based comparisons. Significance levels: *p<.05, **p<.01, ***p<.001.

**Table 5:** Pairwise Contrasts for Score Differences by the Decision to Read.


PRIOR KNOWLEDGE	EXPERIMENT 1				EXPERIMENT 2			
	
	DIFF	SE	*t*	*p*	DIFF	SE	*t*	*p*

0%	–0.54	0.20	–2.71	.007**	–0.97	0.16	–6.05	<.001***

33%	–0.64	0.08	–7.78	<.001***	–0.76	0.08	–8.92	<.001***

67%	–0.39	0.06	–6.56	<.001***	–0.41	0.06	–7.03	<.001***

100%	–0.05	0.06	–0.85	.397	–0.04	0.06	–0.68	.499


*Note*. Values reflect estimated marginal mean differences in pretest-to-posttest scores between different decisions– reading or skipping– for each prior knowledge condition. Negative values indicate larger score gains in the “read” group. Significance levels: *p<.05, **p<.01, ***p<.001.

**Table 6:** Pairwise Contrasts for Score Differences Between Groups.


PRIOR KNOWLEDGE	EXPERIMENT 1				EXPERIMENT 2			
	
	DIFF	SE	*t*	*p*	DIFF	SE	*t*	*p*

0%	0.47	0.21	2.21	.027*	0.39	0.17	2.26	<.024*

33%	0.10	0.10	1.02	.310	0.56	0.10	5.83	<.001***

67%	0.08	0.08	1.08	.281	0.27	0.07	3.64	<.001***

100%	0.06	0.08	0.71	.477	0.11	0.07	1.48	.138


*Note*. Values reflect estimated marginal mean differences in pretest-to-posttest scores between experimental and control groups for each prior knowledge condition. Positive values indicate larger score gains in the experimental group. Significance levels: *p<.05, **p<.01, ***p<.001.

Before conducting our main analysis, we excluded participants who made uniform decisions (as we preregistered; see Online Supplementary for the data analysis considering all participants) to read across the experiment (i.e., always choosing to read or always choosing to skip, *n* = 118 in the experimental group and *n* = 148 in the control group). We excluded these participants as they did not add any variance to our *probability to read* variable. In total, *n* = 100 participants in the control group (40.3%) and *n* = 76 in the experimental group (30.0%) chose to read all six chapters. In contrast, *n* = 48 (19.4%) and *n* = 42 (16.6%) participants in the control and experimental groups, respectively, skipped all chapters. The proportion of participants who read at least one chapter was 80.6% in the control group and 83.4% in the experimental group. After we applied this exclusion criterion, the remaining sample considered *n* = 235 participants (*n* = 135 in the experimental group and *n* = 100 in the control group).

#### Illustrating Knowledge Gaps Increases Information-Seeking Behavior

Our first analysis examined participants’ probability to read as a function of their prior knowledge and group. Results showed a significant interaction between prior knowledge and group for both the first (*β* = 38.71, *z* = 7.75 *p* < .001) and the second polynomial term (*β* = 9.58, *z* = 2.04, *p* = .042). This indicated a difference between groups in the relationship between prior knowledge and the probability to read. We expected an inverted U-shaped relationship between prior knowledge and the probability to read in the experimental group. However, the curve for the experimental group rather suggested a non-linear and negative relationship between prior knowledge and reading behavior with relatively similar average values for 0% prior knowledge and 33% prior knowledge (see Figure 2a). Participants in the control group had relatively similar average values across prior knowledge conditions (also see pairwise comparisons reported below).

As expected, the pairwise comparisons between the two groups at each prior knowledge condition revealed that participants in the experimental group had a significantly higher probability to read than the control group at prior knowledge levels of 33%, and a significantly lower probability to read at 100% prior knowledge (see Figure 2a and Table 3). In addition, and against our expectations, we observed that participants in the experimental group had a significantly higher probability to read than the control group at prior knowledge levels of 0%, and the results did not reveal a significant difference between groups at 67% prior knowledge.

The pairwise comparisons within each group revealed significant differences in the experimental group between 0% and 100%, 33% and 100%, and 67% and 100%, in line with our expectations. Against our expectations, we observed a significant difference between 33% and 67% prior knowledge (see Table 4). In the control group, as expected, we observed no significant differences in the probability to read between prior knowledge conditions.

#### Knowledge Gains as a Function of Prior Knowledge and Decision to Read

As expected, the pairwise comparisons indicated significant differences in knowledge gains between reading and skipping at the three prior knowledge conditions of 0%, 33% and 67%. The comparison at 100% prior knowledge was not significantly different (see Figure 2b and Table 5).

#### Knowledge Gains as a Function of Prior Knowledge and Group

Results of knowledge gains as a function of prior knowledge and group are shown in Figure 2c. Pairwise comparisons between groups did not show significant results, except for the 0% prior knowledge condition (see Table 6). This indicated that the score differences from pretest to posttest were higher in the experimental group only for very large knowledge gaps. As such, this result was not in line with our expectation that a significantly higher probability to read, as observed for the 33% prior knowledge condition, is accompanied with significantly increased knowledge gains.

### Additional Analyses

**Figure 3:**
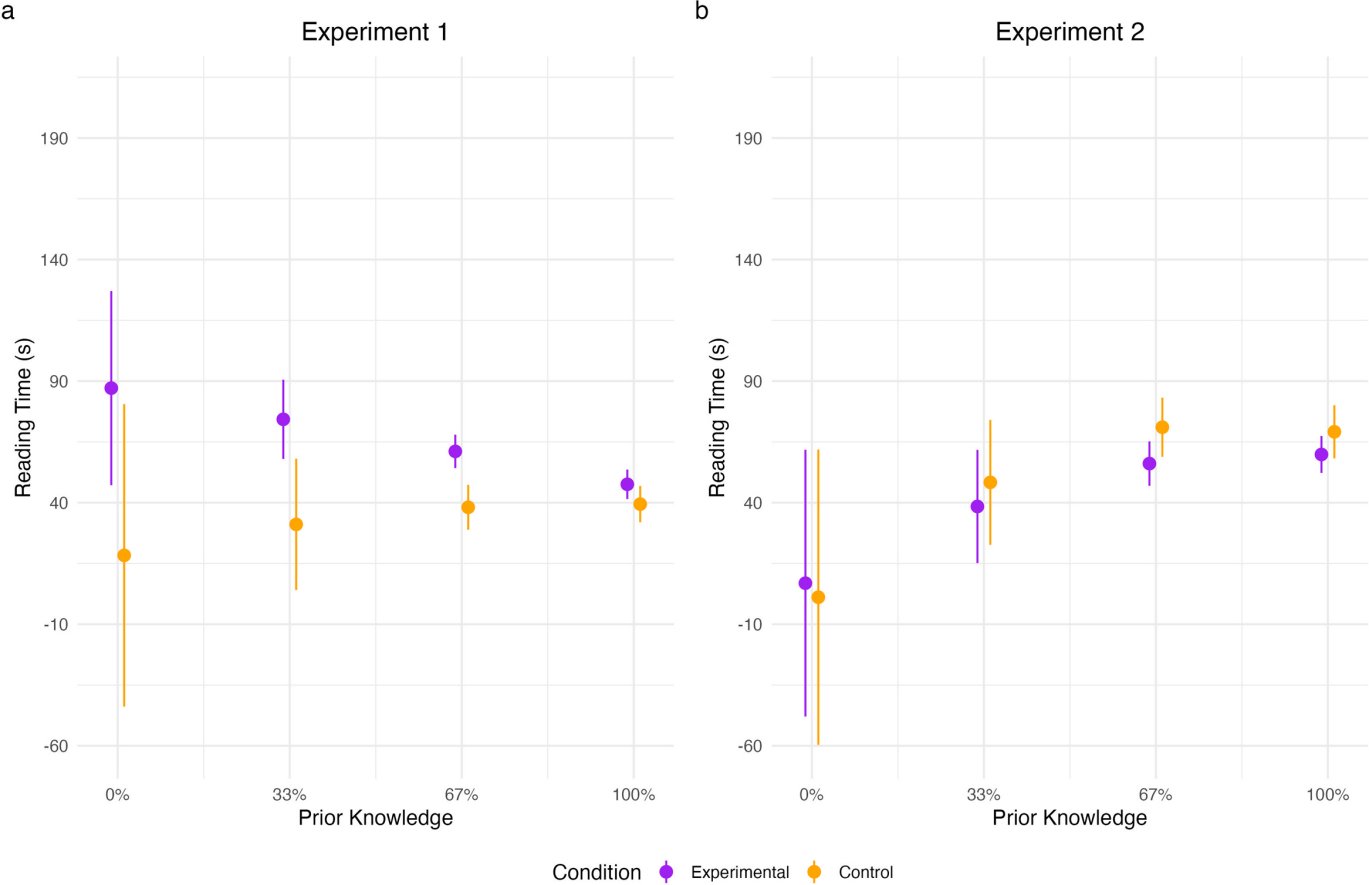
Plots display reading time in seconds by prior knowledge and group.

We further examined reading time as a function of prior knowledge and group. Results for pairwise contrasts between groups for each prior knowledge condition are shown in Table 7 and visualized in Figure 3. In Experiment 1, results revealed a significantly longer reading times in the experimental group compared to the control group at 67% prior knowledge.

**Table 7:** Pairwise Contrasts for Reading Times Between Groups. *Note*. Values reflect estimated marginal mean differences in reading time (in seconds) between experimental and control groups at each prior knowledge level. Positive differences (Diff) indicate longer reading times in the experimental group. Significance levels: *p<.05, **p<.01, ***p<.001.


PRIOR KNOWLEDGE	EXPERIMENT 1				EXPERIMENT 2			
	
	DIFF	SE	*t*	*p*	DIFF	SE	*t*	*p*

0%	68.80	73.90	0.93	.352	5.77	81.8	0.07	.944

33%	43.22	31.50	1.37	.171	–9.93	34.7	–0.29	.775

67%	23.00	11.50	2.00	.046*	–14.95	15.2	–0.98	.326

100%	8.15	9.61	0.85	.397	–19.31	13.3	–0.70	.484


**Figure 4:**
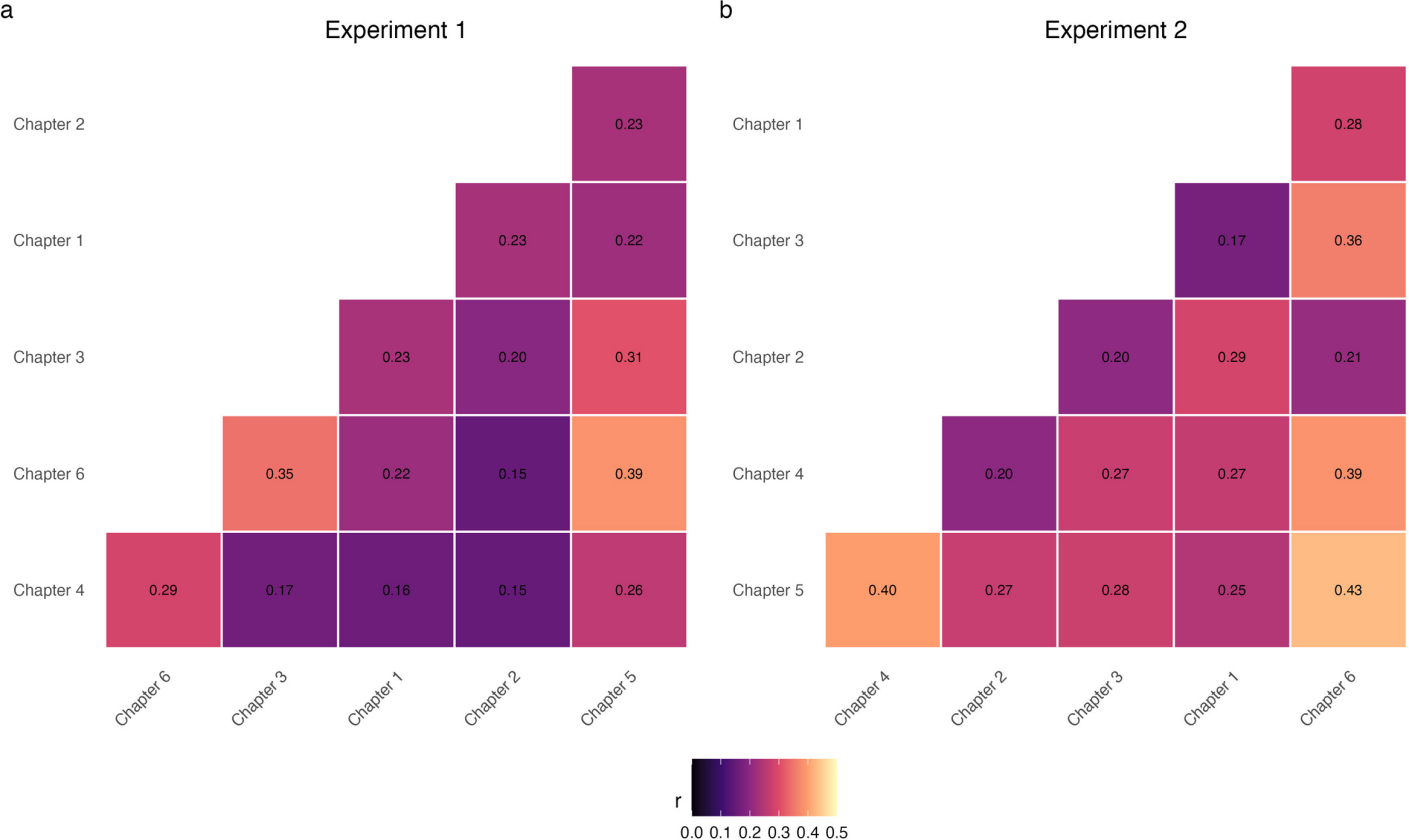
Heatmaps display correlations for prior knowledge between chapters for Experiment 1 and Experiment 2.

Finally, we examined how strongly prior knowledge was correlated across chapters. Therefore, we calculated correlation matrices and constructed heatmaps for each experiment (Figure 4). Across all experiments, prior knowledge showed correlations between r = .15 and r = .43 between chapters. Following common guidelines (Cohen, 1988), our data shows small-to-medium positive correlations of prior knowledge.

### Discussion

The aim of Experiment 1 was to test whether knowledge gap illustrations spark curiosity. First, we observed significantly different regression curves for the two groups, suggesting that illustrating knowledge gaps affected the experimental group differently than the control group for whom we did not illustrate knowledge gaps. Importantly, we also observed this in the pilot experiment (see Online Supplementary). Further inspection of the contrasts between groups indicated a significant difference between the two groups at 0% and 33% prior knowledge, with a higher probability to read in the experimental group than the control group. While we expected high information-seeking for moderate knowledge gap illustrations (33% prior knowledge), we did not expect it at 0% prior knowledge. However, we only observed a significant difference at 33% prior knowledge in the pilot experiment and did not observe a significant difference at 0% prior knowledge in the pilot experiment. Moreover, only few participants had 0% prior knowledge (see Table 2). Therefore, the significant difference at 0% prior knowledge has to be interpreted with caution. In addition, and as expected, the experimental group had a significantly lower probability to read than the control group at 100% prior knowledge. This supports our hypothesis that curiosity vanishes when illustrating that full knowledge exists. Finally, we observed no differences in reading probabilities between the two groups at 67% prior knowledge, even though we expected such differences not only at 33% prior knowledge but also at 67% prior knowledge. Nevertheless, the differing regression curves reliably found in the pilot experiment and Experiment 1 as well as the robust finding of a significant difference at 33% prior knowledge and 100% prior knowledge largely support our hypothesis that illustrating moderate knowledge gaps with lower knowledge spark curiosity and conversely, that illustrating that full knowledge exists diminishes curiosity.

We substantiated these results by also examining knowledge gains across groups for conditions where knowledge gaps existed (0%, 33%, and 67%). We observed (in the pilot experiment and Experiment 1) that information-seeking behavior was accompanied by significantly increased knowledge gains across groups. However, between-group comparisons at each prior knowledge condition were not in line with our expectations. We expected larger knowledge gains in the experimental group for prior knowledge conditions where we also observed elevated reading probabilities compared to the control group. However, we only observed a significant difference in knowledge gains at the 0% prior knowledge condition in Experiment 1, but not at the 33% condition where we observed higher reading probabilities in the experimental group. Note that we also did not observe significantly larger knowledge gains at 33% prior knowledge in the pilot experiment.

We reasoned that increasing the utility of gaining knowledge may modulate the results towards larger knowledge gains for the experimental group than the control group. In addition, we hypothesized that increasing the utility of gaining information should results in a robust difference in the probability to read at 0% prior knowledge, favoring the experimental group. We therefore intended to increase the utility of gaining knowledge in Experiment 2.

## Experiment 2

In Experiment 2, we sought to examine whether increasing the utility for reading chapters affected participants’ probability to close large knowledge gaps (i.e., 0% prior knowledge). In particular, we hypothesized the inclusion of a utility cue in the instructions (i.e., telling participants that a posttest would follow the reading phase) to increase the probability to read as well as increase knowledge gains in the experimental group in comparison to the control group, specifically for large knowledge gaps (i.e., 0% prior knowledge).

In addition, we had the same expectations as in Experiment 1. We expected that illustrating moderate knowledge gaps (i.e., 33% and 67%) would result in an elevated probability to read (i.e., information-seeking behavior). We further expected increased information-seeking behavior to result in heightened knowledge gains in the experimental group.

We preregistered Experiment 2 via AsPredicted (AsPredicted #222,391 on 04/10/2025 at 01:44 AM [PT]). As in Experiment 1, we only included participants with variation in their reading behavior in our final analysis. Importantly, we preregistered this inclusion criteria and also report an analysis considering all participants in the Online Supplementary. As we did for the power simulation for Experiment 1, we ran 100 simulations and targeted an alpha level of *α* = .05 at a sample size of *n* = 200 for the first power simulation on the interaction effect of prior knowledge and group on the *probability to read*. The power simulation calculated a power of 99% with a 95% confidence interval (CI) between 94.6% and 100%. This calculation was based on the effect size *β* = 11.70 for the quadratic term of the interaction that we found in our pilot experiment for Experiment 2 (see the Online Supplementary). As we expected to exclude half of our participants, we conservatively selected a sample size of *n* = 500 participants.

### Method

#### Participants

We collected data from *n* = 511 participants (age range = 18–45; mean age = 25.1; 255 females; 256 males) online via Prolific. Participants obtained US$2 for participating in the experiment. The experiment was run in English. The experiment lasted for 13.3 minutes on average (*SD* = 9.1). All participants gave consent that their anonymous data could be stored and published before the start of the study. Participants were told that they could stop the study at any time.

#### Stimuli, Procedure, and Data Analysis

The stimulus material, procedure, and data analysis were the same as in Experiments 1, with one key modification in the procedure. In the instructions—after the pretest and before beginning the reading part of the experiment—we explicitly informed participants that they will take the same test again at the end. The instructions read: “Next, you will read an article that discusses various aspects of elephant conservation in Botswana. The article is divided into sections, each covering a specific aspect of the topic. After each section of the article, you will be asked whether you want to continue reading the next section or skip it. Finally, you will take the same quiz as before once again to measure changes in your knowledge after reading the article.” As in Experiment 1, we randomized the order in which chapters were presented.

#### Transparency and Openness

We have documented all aspects of the study in accordance with transparency standards, including criteria for data exclusion, sample size determinants (i.e., power analyses), and all experimental manipulations. The complete analysis code, dataset and stimulus materials are available at osf.io/bk9c3. Experiment 2 was preregistered via AsPredicted (AsPredicted #222,391 on 04/10/2025 at 01:44 AM [PT]).

### Results

Figure 2d, 2e and 2f depict the results of Experiment 2. BIC comparisons revealed that our preregistered model explained our data best (see Table 1). Table 2 shows the distributions of participants, trials, and average trials per participant across prior knowledge conditions and group. As in Experiment 1, we again saw relatively few participants in the 0% prior knowledge conditions of both groups. Results of the pairwise comparisons are listed in Table 3 (pairwise contrasts in the probability to read between groups), Table 4 (pairwise contrasts within groups for each pair of prior knowledge conditions), Table 5 (pairwise contrasts for knowledge gains by decision to read), and Table 6 (pairwise contrasts for knowledge gains between groups).

As in Experiment 1 and as preregistered, we excluded participants who always chose to read or always chose to skip, *n* = 120 in the experimental group and *n* = 162 in the control group; but see the Online Supplementary for the results considering all participants). In Experiment 2, *n* = 130 participants in the control group (51.0%) and *n* = 89 in the experimental group (43.8%) chose to read all six chapters. However, *n* = 32 (12.5%) participants in the control and *n* = 31 (12.1%) in the experimental group skipped all chapters. The proportion of participants who read at least one chapter was almost the same across groups (control: 87.5%, experimental: 87.9%). This exclusion left *n* = 229 participants remaining for our analysis (*n* = 136 in the experimental group and *n* = 93 in the control group).

#### Illustrating Knowledge Gaps Increases Information-Seeking Behavior

The results showed a significant interaction between prior knowledge and group for both the first (*β* = 48.11, *z* = 8.83 *p* < .001) and the second polynomial term (*β* = 30.23, *z* = 6.31, *p* < .001). These interactions suggest that reading decisions differed by group. This, analogue to Experiment 1, posits a different relationship between the slopes of both groups as we expected (see Figure 2d). In particular, we observed an overall downward sloped curve in the experimental condition, with the highest point representing the 33% prior knowledge condition. As expected, the control group’s reading behavior indicated a relatively flat line, suggesting that prior knowledge in the control group did not influence their decision to read.

As expected, pairwise comparisons between groups revealed a significantly higher reading probability in the experimental group compared to the control group at 33% and 67% prior knowledge and a significantly lower one at 100% prior knowledge (see Figure 2d and Table 3). However, we did not find the hypothesized increased probability to read in the experimental group compared to the control group at 0% prior knowledge.

The pairwise comparisons on prior knowledge conditions within groups on the probability to read did not show any significant contrasts in the control group. We found a significant effect comparing 0% and 33% in the experimental group, which we had not seen in Experiment 1, indicating higher reading probabilities at 33% compared to 0% prior knowledge (see Table 4). This meant that all contrasts within the experimental group indicated significant differences, except for the contrast between 0% and 67% prior knowledge. Together, these results show that curiosity peaks at 33%, but are somewhat contradicting our expectations, as we anticipated the largest probability to read at 0% prior knowledge and no significant difference between 33% and 67% prior knowledge.

#### Knowledge Gains as a Function of Prior Knowledge and Decision to Read

Across groups and as expected, participants improved more when choosing to read than when skipping chapters when having 0%, 33%, and 67% prior knowledge (see Figure 2e and Table 5).

#### Knowledge Gains as a Function of Prior Knowledge and Group

Contrast comparisons indicated significantly higher knowledge gains in the experimental group than the control group at 0%, 33%, and 67% prior knowledge (see Figure 2f and Table 6). This finding aligns with our hypothesis that an increased probability to read should be accompanied by increased knowledge gains, but only for the 33% and 67% prior knowledge conditions. Unexpectedly, for the 0% prior knowledge condition, the experimental group showed significantly higher knowledge gains than the control group, despite no observed difference between the groups in the preceding decision to read.

#### Additional Analyses

Results for reading time as a function of prior knowledge are shown in Figure 3 and Table 7. In Experiment 2, the results did not reveal any significant differences in pairwise comparisons.

Finally, the correlation matrices of Experiment 2 indicated small-to-medium positive correlations of prior knowledge between chapters. This suggests that prior knowledge was not strongly correlated across chapters.

### Discussion

The aim of Experiment 2 was to test whether increasing the utility of gaining knowledge would strengthen the effects of knowledge gap illustrations on curiosity, particularly for large knowledge gaps (0% prior knowledge). Adding a utility cue in the instructions was intended to increase the probability to read and improve knowledge gains in the experimental group compared to the control group, especially at 0 % prior knowledge.

We observed significantly different regression curves for the two groups, consistent with Experiment 1 and the pilot experiments. Pairwise contrasts revealed a significantly higher probability to read in the experimental group at 33% prior knowledge and a significantly lower probability to read at 100% prior knowledge, replicating these effects from Experiment 1. We also observed a significantly higher probability to read in the experimental group at 67% prior knowledge. However, contrary to our hypothesis, we found no significant group difference in the probability to read at 0% prior knowledge. Thus, even with the added utility cue, we did not replicate the 0% prior knowledge effect seen in the second pilot experiment.

Within-group comparisons supported these observations. In the control group, there were no significant differences between prior knowledge conditions. In the experimental group, all contrasts were significant except between 0% and 67% prior knowledge. This pattern did not match our prediction that 0% prior knowledge would yield the highest probability to read and differ significantly from all other conditions. Instead, the highest reading probability was again observed at 33% prior knowledge, suggesting that curiosity peaked at moderate knowledge gaps.

As in Experiment 1, reading was associated with significantly greater knowledge gains at 0%, 33%, and 67% prior knowledge across groups. Between-group contrasts showed that the experimental group outperformed the control group at all three levels. While this was in line with our prediction for 33% and 67% prior knowledge, the finding at 0% prior knowledge was unexpected, as it occurred without a preceding increase in the probability to read. This dissociation suggests that the utility cue may have enhanced learning outcomes for 0% prior knowledge, especially among those who chose to read in the experimental group, even though it did not increase the decision to do so.

Overall, Experiment 2 provides mixed support for the hypothesis that increasing utility boosts curiosity for large knowledge gaps. Although the utility cue did not increase the probability to read when prior knowledge was 0%, it was associated with higher knowledge gains at this level. The replicated effects at 33% prior knowledge, and the increased probability at 67% prior knowledge—which may be due to the utility cue—along with the consistent reduction at 100% prior knowledge, reinforce the conclusion that highlighting moderate knowledge gaps—primarily those with lower knowledge— reliably sparks curiosity, whereas illustrating full knowledge diminishes it.

## General Discussion

We investigated whether explicitly illustrating knowledge gaps would spark curiosity, measured as information-seeking behavior, and whether this behavior would lead to increased knowledge gains. In two preregistered experiments (each preceded by an identical pilot study with about 300 participants, respectively), participants repeatedly decided whether to read or skip chapters from an article. We estimated prior knowledge using a pretest for each chapter and knowledge gaps were operationalized as the percentage of incorrectly answered items. In the experimental condition, we showed participants their chapter-specific knowledge gaps before each decision; in the control condition, we provided no such information. In Experiment 2, we increased the perceived utility of reading by informing participants that a posttest would follow. Based on the information-gap theory and the rational account of curiosity, we had predicted that (a) illustrating moderate gaps at both 33% and 67% prior knowledge would increase curiosity compared to the control group, (b) that illustrating full knowledge (100%) would diminish it, and (c) that large gaps (0%) might be more sensitive to utility manipulations.

Across both experiments, we observed three key findings. First, illustrating moderate knowledge gaps (33% prior knowledge) increased the probability of reading relative to the control group, supporting the information-gap theory’s prediction that curiosity peaks at moderate gaps (cf. Kang et al., 2009; Loewenstein, 1994). Second, illustrating full prior knowledge (100%) reduced the probability of reading, consistent with the idea that curiosity diminishes when no gap exists (cf. Loewenstein, 1994; Metcalfe et al., 2023). Third, participants who chose to read generally gained more knowledge than those who skipped, except when they already had complete knowledge, supporting the hypothesis that the information-seeking behavior generally led to knowledge gains.

The two experiments diverged in the effects for large gaps (0% prior knowledge). In Experiment 1, illustrating large gaps unexpectedly increased reading probability in the experimental group relative to the control group. In Experiment 2, the utility manipulation did not produce a significant difference in reading probability for large gaps in the experimental group compared to the control group. However, in both experiments, the experimental group gained more knowledge than the control group at 0% prior knowledge, even when we did not observe higher reading rates. This discrepancy likely reflects low statistical power in the 0% condition due to small sample sizes. Future research could address this limitation by selecting a topic for which participants typically have very low prior knowledge but perceive high utility in gaining it. This approach could increase the number of participants and trials per participant in the 0% prior knowledge condition, thereby improving the power to detect effects in this critical range.

Together, these findings suggest that highlighting moderate knowledge gaps (33%) effectively stimulates curiosity, whereas explicitly indicating full prior knowledge diminished curiosity. Notably, our findings for the 67% prior knowledge condition did not align fully with our predictions. We had anticipated that both moderate gap conditions (33% and 67%) would similarly increase curiosity and information-seeking relative to the control group. While this pattern emerged for 67% prior knowledge in Experiment 2, it was absent in Experiment 1. One possible explanation is that the higher-moderate gap lies closer to the point where curiosity begins to decline as the knowledge gap narrows, making it more sensitive to contextual influences such as the presence of a utility cue. This interpretation is consistent with the rational account of curiosity, which predicts that smaller gaps may require higher perceived utility to prompt information-seeking. Future studies could test this more directly by systematically varying both the magnitude of the gap and the utility context within a single design. Nevertheless, our results provide further support for the information-gap theory positing that curiosity peaks at moderate knowledge gaps.

Our work extends existing theories in two key ways. First, prior studies on the information-gap theory have typically estimated knowledge gaps indirectly, often relying on measures such as confidence self-ratings (e.g., Dubey & Griffiths, 2020; Kang et al., 2009; Spitzer et al., 2024). These approaches may have made gaps salient only implicitly, leaving it unclear whether explicitly presenting a learner’s knowledge gap—beyond such indirect cues—would spark curiosity. We addressed this question directly by showing/not showing participants their chapter-specific knowledge gaps before each decision, and we found that showing moderate knowledge gap illustrations (33%) increased information-seeking behavior compared to not showing such knowledge gaps.

Second, the information-gap theory was originally developed to account for discrete, “one-and-done” curiosity events, where curiosity is resolved immediately upon receiving an answer. In contrast, we embedded curiosity elicitation in a naturalistic reading context (see also Schumacher et al. (2024, 2025)), using chapters on subtopics about elephant conservation in Botswana^3^. This design extends trivia-question tasks demonstrating that theoretical predictions about curiosity hold in richer, more ecologically valid learning settings.

Beyond curiosity research, our findings can also be related to work on the selection of task difficulty. Previous research suggests that a preference for moderately difficult tasks reflects avoidance of negative affect, such as shame following failure on very easy tasks (Atkinson & Litwin, 1960). Another account by Trope (1975) suggests that selecting moderately difficult tasks is the result of optimizing information gain. Our results are in line with this work, suggesting that participants sought information when knowledge gaps were moderate. This is also consistent with recent integrative accounts linking achievement motivation and curiosity (Ten et al., 2025).

Our findings are in line with previous work by Butler & Winne (1995). They argue that feedback supports learning when it makes discrepancies between current understanding and a desired goal. By explicitly illustrating knowledge gaps before each decision, we highlighted such information. The increased information-seeking behavior at moderate knowledge gaps suggests that visualizing these gaps prompted participants to seek information to close their knowledge gaps.

### Limitations and future research avenues

Our research has several limitations. First, we conducted both experiments on a single topic using one stimulus set, which limits the generalizability of the findings. Future research should examine whether these effects replicate across varied topics, tasks, and domains (e.g., mathematics, history). Second, we did not measure participants’ baseline interest in the topic, which may have influenced engagement independently of the knowledge gap manipulation. Third, our four-level prior knowledge measure (0%, 33%, 67%, 100%) provided only a coarse view of the curiosity–knowledge gap function. Using finer increments (e.g., 10–20%) and ensuring a more balanced distribution of trials across levels could yield a more precise understanding of this relationship. Finally, the pretest itself may have induced curiosity. For example, Law et al. (2016) propose that presenting questions can implicitly elicit curiosity by making knowledge gaps salient. It is therefore possible that the pretest increased curiosity across all groups, and that the effects we observed between groups reflect additional effects on top of this baseline curiosity.

A further limitation concerns the probabilistic structure of the pretest. Each item consisted of one correct answer and three distractors, implying a 25% chance of answering a question correctly by guessing. Consequently, very low pretest scores (i.e., 0%) were relatively unlikely to occur by chance and participants’ might have achieved a pretest score of 33% by chance. This reduced the number of observations in the 0% prior knowledge condition and introduced noise at other prior knowledge levels. Future research should address this issue by reducing guessing probability, for example, by increasing the number of response alternatives. Additionally, a “do not know” answer option could mitigate the possibility of guessing, as participants would not be forced to make a choice.

Our findings are limited by the fact that we did not examine whether illustrating knowledge gaps affected participants’ information-seeking behavior directly, or whether the knowledge gap illustrations influenced other aspects of human cognition, such as participants’ motivation (e.g., achievement goals, self-efficacy) and affective responses, that may then, in turn, influence their decision to search for information and engage with the texts. To illustrate, receiving feedback that one’s current level of knowledge is relatively low may evoke negative emotions that lead one to withdraw from the task (i.e., not engage in information search). Furthermore, previous work elaborated on additional factors influencing people’s curiosity, such as generating predictions (Brod & Breitwieser, 2019) or the ability to make a choice (Jiwa et al., 2021; Murty et al., 2015; Romero Verdugo et al., 2023). Nevertheless, while we encourage future research to test potential alternative pathways, we emphasize that or work is strongly grounded in the rich literature on curiosity identifying knowledge gaps as important factor that sparks curiosity (Berlyne, 1950; Dubey & Griffiths, 2020; Grossnickle, 2016; Gruber et al., 2014; Hsiung et al., 2023; Jach et al., 2024; Kang et al., 2009; Kidd & Hayden, 2015; Kim et al., 2024; Litman, 2005; Loewenstein, 1994; Murayama, 2022; Murayama et al., 2019; van Lieshout et al., 2020; Wilson, 2024; Yagi et al., 2023), for a study using feedback similar to our work, see Metcalfe et al., 2023. Moreover, as noted above, in our experimental paradigm, completing the pretest could potentially enhance the salience of knowledge gaps. However, if our findings were purely driven by the presence of the pretest, then we would not find differences between the experimental group and the control group.

### Conclusion

By explicitly illustrating knowledge gaps, we increased curiosity and, under higher-utility conditions, observed accompanying knowledge gains. We demonstrated that moderate gaps stimulate information-seeking, whereas no gaps suppress it. We examined these effects in a text-based learning context, extending curiosity theory to more naturalistic environments. Overall, our findings support the conclusion that making knowledge gaps explicitly salient can spark curiosity, and they suggest practical avenues for leveraging this mechanism to promote engagement and learning in educational settings.

## Additional File

The additional file for this article can be found as follows:

10.5334/joc.501.s1 Online Supplement. Additional analyses for pilot experiments and full samples as well as the complete stimulus material.

## Data Availability

Raw data and commented analysis scripts are available via the Open Science Framework (anonymized link for peer-review: osf.io/bk9c3).

## Footnotes

Novelty and large knowledge gaps are often assumed to be closely linked. Please note, however, that they are not always equivalent. For instance, a highly novel stimulus—such as visual noise on a screen—can be entirely unfamiliar, yet fails to create a large knowledge gap because it lacks meaningful information and therefore, most likely, also fails to elicit curiosity.

The final group sizes may vary between groups due to the exclusion criterion.

Note that the subtopics did not build upon each other. Knowledge about Chapter 1 was not necessary to answer Chapter 2 and chapters were randomly presented as described in the Methods.

## References

[1] Atkinson, J. W., & Litwin, G. H. (1960). Achievement motive and test anxiety conceived as motive to approach success and motive to avoid failure. The Journal of Abnormal and Social Psychology, 60(1), 52. 10.1037/h0041119 13794971

[2] Bates, D., Mächler, M., Bolker, B., & Walker, S. (2015). Fitting linear mixed-effects models using lme4. Journal of Statistical Software, 67(1), 1–48. 10.18637/jss.v067.i01

[3] Berlyne, D. E. (1950). Novelty and curiosity as determinants of exploratory behaviour. British Journal of Psychology, 41(1), 68. 10.1111/j.2044-8295.1950.tb00262.x

[4] Berlyne, D. E. (1966). Curiosity and exploration: Animals spend much of their time seeking stimuli whose significance raises problems for psychology. Science, 153(3731), 25–33. 10.1126/science.153.3731.25 5328120

[5] Brod, G., & Breitwieser, J. (2019). Lighting the wick in the candle of learning: Generating a prediction stimulates curiosity. NPJ Science of Learning, 4(1), 17. 10.1038/s41539-019-0056-y 31646002 PMC6803639

[6] Burnham, K. P., & Anderson, D. R. (2002). Model selection and multimodel inference: A practical information-theoretic approach. Springer.

[7] Butler, D. L., & Winne, P. H. (1995). Feedback and self-regulated learning: A theoretical synthesis. Review of Educational Research, 65(3), 245–281. 10.3102/00346543065003245

[8] Cantor, J. H., & Cantor, G. N. (1964). Observing behavior in children as a function of stimulus novelty. Child Development, 119–128. 10.2307/1126576 14128800

[9] Cohen, J. (1988). Statistical power analysis for the behavioral sciences. Routledge.

[10] Devine, S., & Otto, A. R. (2022). Information about task progress modulates cognitive demand avoidance. Cognition, 225, 105107. 10.1016/j.cognition.2022.105107 35349871

[11] Dubey, R., & Griffiths, T. L. (2020). Reconciling novelty and complexity through a rational analysis of curiosity. Psychological Review, 127(3), 455. 10.1037/rev0000175 31868394

[12] Fantz, R. L. (1964). Visual experience in infants: Decreased attention to familiar patterns relative to novel ones. Science, 146(3644), 668–670. 10.1126/science.146.3644.668 14191712

[13] Golman, R., Hagmann, D., & Loewenstein, G. (2017). Information avoidance. Journal of Economic Literature, 55(1), 96–135. 10.1257/jel.20151245

[14] Golman, R., & Loewenstein, G. (2018). Information gaps: A theory of preferences regarding the presence and absence of information. Decision, 5(3), 143–164. 10.1037/dec0000068

[15] Green, P., & MacLeod, C. J. (2016). Simr: An r package for power analysis of generalised linear mixed models by simulation. Methods in Ecology and Evolution, 7(4), 493–498. 10.1111/2041-210X.12504

[16] Grossnickle, E. M. (2016). Disentangling curiosity: Dimensionality, definitions, and distinctions from interest in educational contexts. Educational Psychology Review, 28(1), 23–60. 10.1007/s10648-014-9294-y

[17] Gruber, M. J., Gelman, B. D., & Ranganath, C. (2014). States of curiosity modulate hippocampus- dependent learning via the dopaminergic circuit. Neuron, 84(2), 486–496. 10.1016/j.neuron.2014.08.060 25284006 PMC4252494

[18] Hattie, J., & Timperley, H. (2007). The power of feedback. Review of Educational Research, 77(1), 81–112. 10.3102/003465430298487

[19] Hertwig, R., & Engel, C. (2016). Homo ignorans: Deliberately choosing not to know. Perspectives on Psychological Science, 11(3), 359–372. 10.1177/1745691616635594 27217249

[20] Hightow, L. B., Miller, W. C., Leone, P. A., Wohl, D., Smurzynski, M., & Kaplan, A. H. (2003). Failure to return for hiv posttest counseling in an std clinic population. AIDS Education and Prevention, 15(3), 282–290. 10.1521/aeap.15.4.282.23826 12866839

[21] Hsiung, A., Poh, J.-H., Huettel, S. A., & Adcock, R. A. (2023). Curiosity evolves as information unfolds. Proceedings of the National Academy of Sciences, 120(43), e2301974120. 10.1073/pnas.2301974120 PMC10614840 37844235

[22] Jach, H. K., Cools, R., Frisvold, A., Grubb, M. A., Hartley, C. A., Hartmann, J., Hunter, L., Jia, R., de Lange, F. P., Larisch, R., et al. (2024). Individual differences in information demand have a low dimensional structure predicted by some curiosity traits. Proceedings of the National Academy of Sciences, 121(45), e2415236121. 10.1073/pnas.2415236121 PMC11551435 39467138

[23] Jiwa, M., Cooper, P. S., Chong, T. T.-J., & Bode, S. (2021). Choosing increases the value of non-instrumental information. Scientific Reports, 11(1), 8780. 10.1038/s41598-021-88031-y 33888764 PMC8062497

[24] Kang, M. J., Hsu, M., Krajbich, I. M., Loewenstein, G., McClure, S. M., Wang, J. T.-y., & Camerer, C. F. (2009). The wick in the candle of learning: Epistemic curiosity activates reward circuitry and enhances memory. Psychological Science, 20(8), 963–973. 10.1111/j.1467-9280.2009.02402.x 19619181

[25] Kidd, C., & Hayden, B. Y. (2015). The psychology and neuroscience of curiosity. Neuron, 88(3), 449–460. 10.1016/j.neuron.2015.09.010 26539887 PMC4635443

[26] Kim, S., Sakaki, M., & Murayama, K. (2024). Metacognition of curiosity: People underestimate the seductive lure of non-instrumental information. Psychonomic Bulletin & Review, 31(3), 1–12. 10.3758/s13423-023-02404-0 PMC11192831 37932580

[27] Kuznetsova, A., Brockhoff, P. B., & Christensen, R. H. B. (2017). lmerTest package: Tests in linear mixed effects models. Journal of Statistical Software, 82(13), 1–26. 10.18637/jss.v082.i13

[28] Law, E., Yin, M., Goh, J., Chen, K., Terry, M. A., & Gajos, K. Z. (2016). Curiosity killed the cat, but makes crowdwork better. Proceedings of the 2016 CHI Conference on Human Factors in Computing Systems, 4098–4110. 10.1145/2858036.2858144

[29] Lenth, R. V. (2024). Emmeans: Estimated marginal means, aka least-squares means [R package version 1.10.5]. https://CRAN.R-project.org/package=emmeans

[30] Litman, J. (2005). Curiosity and the pleasures of learning: Wanting and liking new information. Cognition and Emotion, 19(6), 793–814. 10.1080/02699930541000101

[31] Litt, A., Reich, T., Maymin, S., & Shiv, B. (2011). Pressure and perverse flights to familiarity. Psychological Science, 22(4), 523–531. 10.1177/0956797611400095 21372324

[32] Loewenstein, G. (1994). The psychology of curiosity: A review and reinterpretation. Psychological Bulletin, 116(1), 75. 10.1037/0033-2909.116.1.75

[33] Lüdecke, D. (2024). Sjplot: Data visualization for statistics in social science [R package version 2.8.16]. https://CRAN.R-project.org/package=sjPlot

[34] Metcalfe, J., Vuorre, M., Towner, E., & Eich, T. S. (2023). Curiosity: The effects of feedback and confidence on the desire to know. Journal of Experimental Psychology: General, 152(2), 464. 10.1037/xge0001284 36048057

[35] Murayama, K. (2022). A reward-learning framework of knowledge acquisition: An integrated account of curiosity, interest, and intrinsic–extrinsic rewards. Psychological Review, 129(1), 175. 10.1037/rev0000349 35099213

[36] Murayama, K., FitzGibbon, L., & Sakaki, M. (2019). Process account of curiosity and interest: A reward- learning perspective. Educational Psychology Review, 31(4), 875–895. 10.1007/s10648-019-09499-9

[37] Murty, V. P., DuBrow, S., & Davachi, L. (2015). The simple act of choosing influences declarative memory. Journal of Neuroscience, 35(16), 6255–6264. 10.1523/JNEUROSCI.4181-14.2015 25904779 PMC4405547

[38] Neath, A. A., & Cavanaugh, J. E. (2012). The bayesian information criterion: Background, derivation, and applications. Wiley Interdisciplinary Reviews: Computational Statistics, 4(2), 199–203. 10.1002/wics.199

[39] Pedersen, T. L. (2024). Patchwork: The composer of plots [R package version 1.3.0]. https://CRAN.R-project.org/package=patchwork

[40] Pisula, W., & Siegel, J. (2005). Exploratory behavior as a function of environmental novelty and complexity in male and female rats. Psychological Reports, 97(2), 631–638. 10.2466/pr0.97.2.631-638 16342593

[41] R Core Team. (2021). R: A language and environment for statistical computing. R Foundation for Statistical Computing. Vienna, Austria. https://www.R-project.org/

[42] Romero Verdugo, P., van Lieshout, L. L., de Lange, F. P., & Cools, R. (2023). Choice boosts curiosity. Psychological Science, 34(1), 99–110. 10.1177/09567976221082637 36287129

[43] Schumacher, A., Kammerer, Y., Scharinger, C., Gottschling, S., Hübner, N., Tibus, M., Kasneci, E., Appel, T., Gerjets, P., & Bardach, L. (2025). How do intellectually curious and interested people learn and attain knowledge? a focus on behavioral traces of information seeking. European Journal of Personality, 08902070241309124. 10.31219/osf.io/6djkr_v2

[44] Schumacher, A., Spitzer, M. W., Jach, H., Kammerer, Y., Scharinger, C., & Bardach, L. (2024). From clicks to curiosity: Exploring self-directed information seeking as a behavioral manifestation of curiosity. 10.31219/osf.io/fj34x 41973775

[45] Shin, D. D., & Kim, S.-i. (2019). Homo curious: Curious or interested? Educational Psychology Review, 31(4), 853–874. 10.1007/s10648-019-09497-x

[46] Smock, C. D., & Holt, B. G. (1962). Children’s reactions to novelty: An experimental study of “curiosity motivation”. Child Development, 631–642. 10.2307/1126663 13914480

[47] Spitzer, M. W. H., Janz, J., Nie, M., & Kiesel, A. (2024). On the interplay of curiosity, confidence, and importance in knowing information. Psychological Research, 88(1), 101–115. 10.1007/s00426-023-01841-9 37278725 PMC10243256

[48] Spitzer, M. W. H., Strittmatter, Y., Marti, M., Schumacher, A., & Bardach, L. (2025). Curiosity overpowers cognitive effort avoidance tendencies. Cognition, 262, 106167. 10.1016/j.cognition.2025.106167 40381339

[49] Sweeny, K., Melnyk, D., Miller, W., & Shepperd, J. A. (2010). Information avoidance: Who, what, when, and why. Review of General Psychology, 14(4), 340–353. 10.1037/a0021288

[50] Tao, G., Branson, B. M., Kassler, W. J., & Cohen, R. A. (1999). Rates of receiving hiv test results: Data from the us national health interview survey for 1994 and 1995. JAIDS Journal of Acquired Immune Deficiency Syndromes, 22(4), 395–400. 10.1097/00126334-199912010-00011 10634202

[51] Ten, A., Oudeyer, P.-Y., Sakaki, M., & Murayama, K. (2025). The curious u: Integrating theories linking knowledge and information-seeking behavior. Open Mind, 9, 1763–1785. 10.1162/OPMI.a.41 41246213 PMC12618012

[52] Trope, Y. (1975). Seeking information about one’s ability as a determinant of choice among tasks. Journal of Personality and Social Psychology, 32(6), 1004. 10.1037/0022-3514.32.6.1004

[53] van Lieshout, L. L., de Lange, F. P., & Cools, R. (2020). Why so curious? quantifying mechanisms of information seeking. Current Opinion in Behavioral Sciences, 35, 112–117. 10.1016/j.cobeha.2020.08.005

[54] Wade, S., & Kidd, C. (2019). The role of prior knowledge and curiosity in learning. Psychonomic Bulletin & Review, 26, 1377–1387. 10.3758/s13423-019-01598-6 31079309

[55] Wansink, B., Cheney, M. M., & Chan, N. (2003). Exploring comfort food preferences across age and gender. Physiology & Behavior, 79(4–5), 739–747. 10.1016/S0031-9384(03)00203-8 12954417

[56] Wickham, H., Averick, M., Bryan, J., Chang, W., McGowan, L. D., François, R., Grolemund, G., Hayes, A., Henry, L., Hester, J., Kuhn, M., Pedersen, T. L., Miller, E., Bache, S. M., Müller, K., Ooms, J., Robinson, D., Seidel, D. P., Spinu, V., Takahashi, K., Vaughan, D., Wilke, C., Woo, K., & Yutani, H. (2019). Welcome to the tidyverse. Journal of Open Source Software, 4(43), 1686. 10.21105/joss.01686

[57] Wilson, T. D. (2024). Curiosity and information-seeking behaviour: A review of psychological research and a comparison with the information science literature. Journal of Documentation, 80(7), 43–59. 10.1108/JD-09-2023-0173

[58] Wisniewski, B., Zierer, K., & Hattie, J. (2020). The power of feedback revisited: A meta-analysis of educational feedback research. Frontiers in Psychology, 10, 487662. 10.3389/fpsyg.2019.03087 PMC6987456 32038429

[59] Yagi, A., FitzGibbon, L., Murayama, K., Shinomori, K., & Sakaki, M. (2023). Uncertainty drives exploration of negative information across younger and older adults. Cognitive, Affective, & Behavioral Neuroscience, 23(3), 809–826. 10.3758/s13415-023-01082-8 37100958

