# Supplementary material for: Knowledge Gap Illustrations Spark Curiosity
Source: J Cogn. 2026 May 7;9(1):28. doi: 10.5334/joc.501 (PMC13155094; doi:10.5334/joc.501)
Supplement: Online Supplement. — Additional analyses for pilot experiments and full samples as well as the complete stimulus material. [file joc-9-1-501-s1.pdf]

Online Supplement

We ran one pilot experiment before we conducted Experiment 1 and 2, respectively. The pilot experiments were conducted to assess whether our experimental design would yield the expected results and to determine our sample sizes for Experiments 1 and 2. Below the analysis for all experiments prior to excluding uniform readers to match our target sample, and the description of the two experiments, please also find the stimulus material applied in all experiments, including instructions as well as the knowledge test implemented as pretest and posttest.

Pilot Experiment 1

Method

Participants

We collected data from 305 participants (age range = 18-45; M = 25.6; 152 females; 153 males) through an online experiment administered on Prolific. The stimulus material was presented in English. Participants received a compensation of US$2. The experiment lasted for 10.9 minutes (SD = 6.6) on average. Prior to the start of the study, all individuals provided informed consent, confirming that their participation was voluntary and that their anonymous data could be stored and published. Participants were informed that they could withdraw from the study at any time.

Stimuli, Procedure and Data Analysis

We used the same stimuli as described in Experiment 1. However, in the pilot experiment to Experiment 1, we only analyzed five instead of six chapters due to a technical error. We did not analyze Chapter 3 ("The Growing Problem of Human-Elephant Conflict"). We also did not randomize the order in which chapters were presented to participants in the pilot experiment. The chapters and their word count are listed in Table S1.

Transparency and Openness

We have documented all aspects of the study in accordance with transparency standards, including criteria for data exclusion and all experimental manipulations. The

Table S1

Chapter ID, Chapter Titles, and Word Count.

Chapter	Chapter Title	Word Count
1	Botswana's Elephant Population and Conservation Challenges	235
2	Historical Decline and Recovery of Elephant Populations	228
3	The Growing Problem of Human-Elephant Conflict	238
4	Policy Approaches to Managing Elephant Populations	294
5	Ethical and Economic Debates on Trophy Hunting and Trade	239
6	Innovative Approaches and the Future of Elephant Conservation	227
**Average**		243.5

Note. Chapters used in the stimulus material for each experiment. We chose similar chapter lengths to make results comparable.

complete analysis code and dataset are available at osf.io/bk9c3. This pilot experiment was not preregistered.

Results

Before conducting our main analysis, we excluded participants who made uniform decisions to read (i.e., always choosing to read or always choosing to skip, n = 76 in the experimental group and n = 110 in the control group). We excluded these participants as they did not add any variance to our *probability to read* variable. In total, n = 64 (42.1%) participants in the control group and n = 38 (24.8%) in the experimental group chose to read all five chapters. In contrast, n = 46 (30.3%) and n = 38 (24.8%) participants in the control and experimental groups, respectively, skipped all chapters. The proportion of participants who read at least one chapter was similar across groups (69.7% in control, 75.2% in experimental). After we applied this exclusion criterion, n = 119 participants remained for further analysis (n = 77 in the experimental group and n = 42 in the control group).

Importantly, we observed relatively few participants in both groups at the 0% prior knowledge condition (see Table S2).

Illustrating Knowledge Gaps Increases Information-Seeking Behavior

Figure 1 illustrates participants probability to read as a function of their prior knowledge and group. Our results indicated a significant interaction between prior knowledge and group for both the first ($\beta = 21.26$, $z = 4.22$, $p < .001$) and the second polynomial term ($\beta = 10.79$, $z = 2.37$, $p = .018$). These interactions suggest that the relationship between prior knowledge and probability to read differed by group: participants in the experimental group exhibited an inverted U-shaped relationship, with the highest probability to read at 33% prior knowledge. However, participants in the control group displayed small positive linear trend, indicating higher reading probabilities with increasing prior knowledge (see Figure **??**a).

Table S3 lists the results comparing contrasts between groups for each prior knowledge condition. These contrast comparisons revealed a significantly higher probability to read in the experimental group compared to the control group at 33% prior knowledge, supporting the hypothesis that moderate knowledge gaps spark curiosity. As expected, the experimental group had a significantly lower probability to read at 100% prior knowledge compared to the control group.

The contrasts between the two groups at 0% and 67% prior knowledge was not significant.

We further examined the difference in the probability to read within each group across all prior knowledge conditions. We list these results in Table S4. We observed no differences between prior knowledge conditions for the control group. Conversely, the results of the experimental group indicated that all contrasts were significantly different from each other, except the comparison between the 0% and 33% and 0% and 67% prior knowledge conditions.

Together, the results of our pilot experiment suggested that illustrating moderate knowledge gaps with lower prior knowledge (33% prior knowledge) significantly increased the probability to read compared to the control group and, at the same time, decreases the probability to read when no knowledge gaps exist (100% prior knowledge). Further contrast comparisons within each group indicated that illustrating knowledge

gaps leads to significant differences between prior knowledge conditions, but we saw no differences in the control group. This suggests that not illustrating knowledge gaps (i.e., the control group) does not lead to differences between the prior knowledge conditions on the probability to read. However, illustrating knowledge gaps resulted in a higher probability to read when knowledge gaps existed (i.e., 0%, 33%, or 67%), compared to when they did not exist (100% knowledge).

Knowledge Gains as a Function of Prior Knowledge and Decision to Read

Figure 1b depicts knowledge gains as a function of prior knowledge and the decision to read (reading vs. skipping) across groups. Note that we made this comparison across groups to test whether reading chapters lead to overall knowledge gains for this prior knowledge condition compared to skipping. The pairwise comparison between reading and skipping at each prior knowledge condition indicated significant differences in knowledge gains between reading and skipping at 0%, 33%, and 67% prior knowledge. The comparison at 100% was not significantly different (see Table S5). As such, our results provide support that reading chapters was accompanied by knowledge gains.

Knowledge Gains as a Function of Prior Knowledge and Group

Figure 1c depicts knowledge gains as a function of prior knowledge and group. The pairwise comparison between the experimental group and the control group indicated significant differences in knowledge gains for 67% prior knowledge, indicating significantly higher knowledge gains in the experimental group compared to the control group (see Table S6). All other pairwise comparisons were not significant. As such, the results were not in line with our expectations of increased knowledge gains in the prior knowledge conditions where we observed an increased probability to read (we observed an increased reading likelihood at 33% prior knowledge and not at 67% prior knowledge).

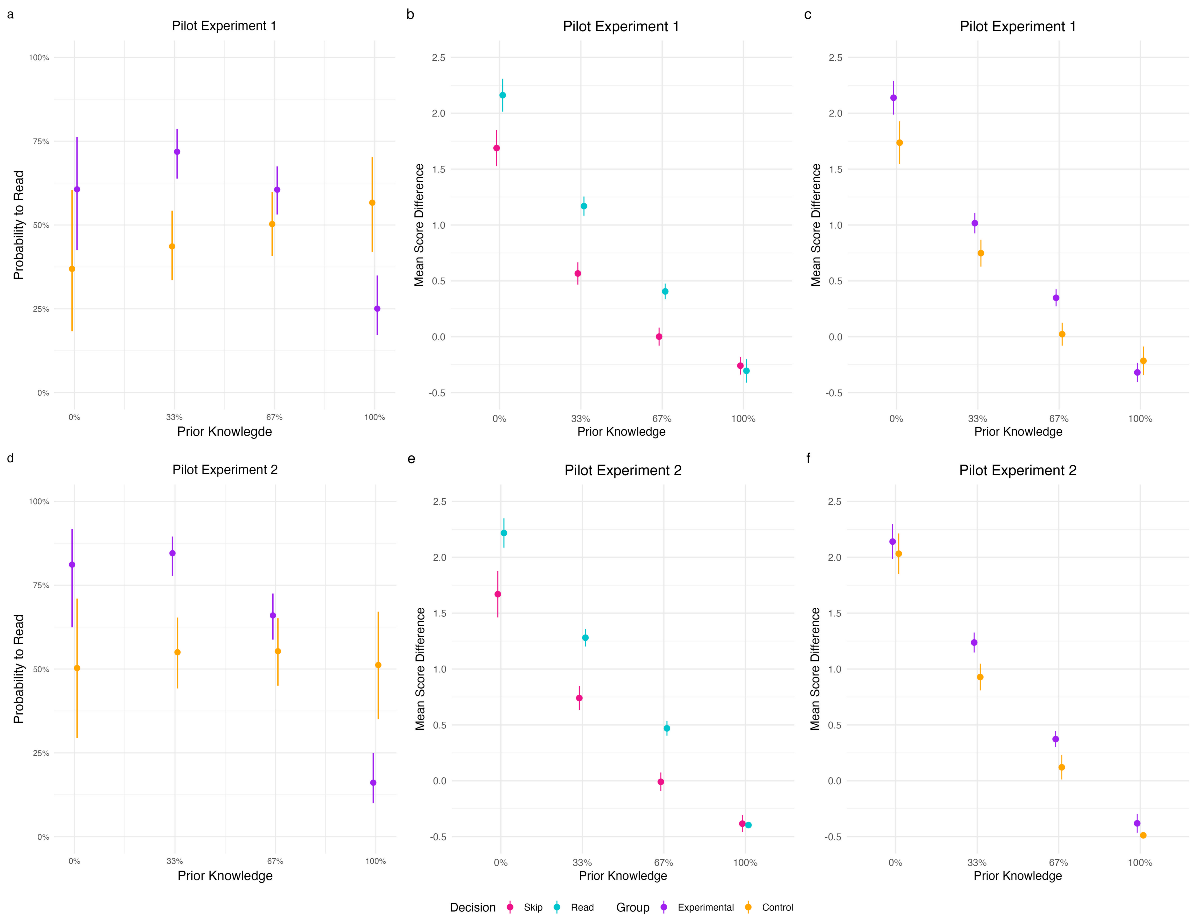

Figure 1

Plots a and d display the probability to read by prior knowledge and group. Plots b and e display the mean score difference by prior knowledge and decision to read. Plots c and f display the mean score difference by prior knowledge and group.

Pilot Experiment 2

Method

Participants

We recruited $n = 297$ participants (age range = 18-45; mean age = 27.3; 149 females; 149 males) via Prolific to participate in this online study. The experiment was conducted in English and participants received US$2 compensation for taking part in the experiment. The experiment lasted for 12.7 minutes on average ($SD = 7.7$). Before beginning the experiment, all participants gave consent that their participation was voluntary and that their anonymous data could be stored and published. Participants

Table S2

Number of Participants, Trials, and Average Trials per Participant by Group and Prior Knowledge for Pilot Experiments.

Experiment	Prior Knowledge	Experimental			Control		
		n(P)	n(T)	M(T/P)	n(P)	n(T)	M(T/P)
**Pilot Experiment 1**	0%	19	30	1.58	15	16	1.07
	33%	56	90	1.61	28	56	2.00
	67%	72	157	2.18	40	88	2.20
	100%	59	108	1.83	27	50	1.85
**Pilot Experiment 2**	0%	20	24	1.20	16	19	1.19
	33%	59	93	1.58	30	58	1.93
	67%	80	183	2.29	36	75	2.08
	100%	65	115	1.77	23	38	1.65

Note. n(P) = number of participants; n(C) = number of trials; M(C/P) = average trials per participant. Values are reported separately for each group (Experimental, Control) across prior knowledge conditions (0%, 33%, 67%, 100%).

Table S3

Pairwise Contrasts for the Probability to Read Between Groups in Pilot Experiments.

Prior Knowledge	Pilot Experiment 1				Pilot Experiment 2			
	OR	SE	z	p	OR	SE	z	p
0%	2.63	1.63	1.56	.119	4.25	2.80	2.20	.028*
33%	3.30	0.97	4.07	<.001***	4.47	1.39	4.80	<.001***
67%	1.52	0.38	1.66	.097	1.57	0.41	1.72	.086
100%	0.26	0.10	-3.48	<.001***	0.18	0.08	-3.88	<.001***

Note. Odds Ratios (*OR*) reflect the contrast: Experimental / Control for each prior knowledge condition. Values are based on estimated marginal means from hierarchical logistic regression models. z-values and p-values correspond to model-based comparisons. Significance levels: $^*p < .05$, $^{**}p < .01$, $^{***}p < .001$.

were told that they could stop the study at any time without giving a reason.

Stimuli, Procedure and Data Analysis

The stimulus material was the same as in Experiment 2. We excluded the same chapter as we did in the first pilot experiment from our analysis due to the same technical issues. Thus, we included five chapters in our analyses. As in the first pilot experiment, this pilot experiment also featured the same order of chapters for each participant.

Transparency and Openness

We documented all aspects of the study in accordance with transparency standards, including criteria for data exclusion, and all experimental manipulations. The complete

Table S4

Pairwise Contrasts Between Prior Knowledge Conditions Within Groups in Pilot Experiments.

Group	Prior Knowledge Contrast	Pilot Experiment 1				Pilot Experiment 2			
		OR	SE	*z*	*p*	OR	SE	*z*	*p*
**Experimental**	0% vs 33%	0.60	0.19	-1.57	.395	0.79	0.30	-0.62	.925
	0% vs 67%	1.00	0.43	0.01	>.999	2.22	1.17	1.50	.436
	0% vs 100%	4.61	2.04	3.45	.003**	22.36	12.70	5.48	<.001***
	33% vs 67%	1.66	0.25	3.45	.003**	2.82	0.53	5.48	<.001***
	33% vs 100%	7.63	2.67	5.81	<.001***	28.42	12.00	7.91	<.001***
	67% vs 100%	4.59	1.23	5.67	<.001***	10.09	3.15	7.41	<.001***
**Control**	0% vs 33%	0.76	0.33	-0.65	.917	0.83	0.34	-0.46	.968
	0% vs 67%	0.58	0.33	-0.96	.773	0.82	0.44	-0.37	.982
	0% vs 100%	0.45	0.24	-1.48	.448	0.97	0.51	-0.07	.999
	33% vs 67%	0.77	0.14	-1.48	.448	0.99	0.17	-0.07	.999
	33% vs 100%	0.59	0.24	-1.31	.560	1.17	0.51	0.35	.985
	67% vs 100%	0.77	0.25	-0.79	.857	1.80	0.41	0.47	.965

Note. Odds Ratios (*OR*) reflect the contrasts between prior knowledge conditions within each group (Experimental / Control). Values are based on estimated marginal means from hierarchical logistic regression models. *z*-values and *p*-values correspond to model-based comparisons. Significance levels: **p* < .05, ***p* < .01, ****p* < .001.

Table S5

Pairwise Contrasts for Score Differences by the Decision to Read in Pilot Experiments.

Prior Knowledge	Pilot Experiment 1				Pilot Experiment 2			
	Diff	SE	*t*	*p*	Diff	SE	*t*	*p*
0%	-0.47	0.21	-2.29	.023*	-0.55	0.24	-2.30	.022*
33%	-0.60	0.12	-4.98	<.001***	-0.54	0.12	-4.42	<.001***
67%	-0.40	0.09	-4.26	<.001***	-0.48	0.09	-5.10	<.001***
100%	0.05	0.12	0.39	.700	0.01	0.14	0.09	.926

Note. Values reflect estimated marginal mean differences in pretest-to-posttest scores between different decisions–reading or skipping– for each prior knowledge condition. Negative values indicate larger score gains in the "read" group. Significance levels: **p* < .05, ***p* < .01, ****p* < .001.

analysis code and dataset can be found at osf.io/bk9c3. This pilot experiment was not preregistered.

Results

We excluded participants who decided to either always read or always skip ($n = 176$ in total; $n = 66$ in the experimental group and $n = 110$ in the control group). In

Table S6

Pairwise Contrasts for Score Differences Between Groups in Pilot Experiments.

Prior Knowledge	Pilot Experiment 1				Pilot Experiment 2			
	Diff	SE	*t*	*p*	Diff	SE	*t*	*p*
0%	0.40	0.24	1.65	.099	0.11	0.24	0.45	.652
33%	0.27	0.15	1.78	.076	0.31	0.15	2.07	.039*
67%	0.33	0.13	2.55	.011*	0.25	0.13	1.94	.053
100%	-0.10	0.16	-0.67	.502	0.11	0.16	0.66	.512

Note. Values reflect estimated marginal mean differences in pretest-to-posttest scores between experimental and control groups for each prior knowledge condition. Positive values indicate larger score gains in the experimental group. Significance levels: $*p < .05$, $**p < .01$, $***p < .001$.

Experiment 2a, $n = 96$ participants in the control group (64.9%) and $n = 49$ in the experimental group (32.9%) chose to read all five chapters. In contrast, $n = 14$ (9.5%) in the control group and $n = 17$ (11.4%) participants in the experimental group skipped all chapters.

The proportion of participants who read at least one chapter was again similar across groups (90.5% in control, 88.6% in experimental). The final sample after applying this exclusion criterion consisted of $n = 121$ participants (experimental group: $n = 83$; control group: $n = 38$).

The second pilot experiment again showed the least amount of trials in the 0% prior knowledge condition by a substantial margin (see Table S2).

Illustrating Knowledge Gaps Increases Information-Seeking Behavior

Both, the first ($\beta = 27.38$, $z = 5.06$, $p < .001$) and second ($\beta = 11.70$, $z = 2.44$, $p = .015$) polynomial term significantly showed significant interactions of prior knowledge with the group variable. These two interactions suggest a negative relationship between prior knowledge and the probability to read for the experimental group. Conversely, participants in the control group showed relatively stable reading probabilities regardless of prior knowledge.

Pairwise comparisons showed a significantly higher probability to read in the experimental group compared to the control group at 0% and 33% prior knowledge and a significantly lower probability to read at 100% prior knowledge (also see Figure 1 and

Table S3).

Pairwise contrasts of prior knowledge within groups on the probability to read revealed no significant differences in the control group. Yet, in the experimental group, all pairwise contrasts indicated significant differences, except for the contrast between 0% and 33% as well as between 0% and 67% (see Table S4).

Knowledge Gains as a Function of Prior Knowledge and Decision to Read.

Pairwise comparisons investigating knowledge gains by the decision to read revealed significantly stronger knowledge gains when reading compared to skipping for 0%, 33% and 67% prior knowledge (see Figure 1, Table S5).

Knowledge Gains as a Function of Prior Knowledge and Group

Pairwise comparisons between groups showed a significant difference in score gains at 33% prior knowledge. This indicates that the experimental group improved significantly more than the control group at 33% prior knowledge. Results are displayed in Figure 1 and Table S6.

Additional Analyses

Order Effects and Trajectories of Deciding to Read

We additionally examined whether chapter order influenced the probability to read (also see Figure S??). To do this, we calculated a model with the effects of both chapter position and group as well as their interaction on the probability to read. We implemented a median split for chapter order (i.e., chapters 1-3 and chapters 4-6). Results were consistent across the two main experiments. We found a main effect of chapter position on the probability to read, indicating that the further along participants got in the experiment, the less likely they were to decide to read (Experiment 1: β = -1.10, z = -7.20, $p < .001$; Experiment 2: β = -0.76, z = -5.12, $p <$ .001). Additionally, results showed a significant interaction term with group in both main experiments (Experiment 1: β = -0.71, z = -2.96, p = .003; Experiment 2: β = -1.06, z = -4.35, $p < .001$).

Results from Analysis Containing All Participants (Pilot Experiments)

Figure 2 shows the plotted probability to read by group, score difference by decision to read and score difference by group for both pilot experiments and both main experiments. Plots a, b, c and g, h, i show results for the two pilot experiments, plots d, e, f and j, k, l illustrate results for the two main experiments. Plots were calculated using the full sample before exclusion of uniform readers. Table S7 displays Number of Trials, number of participants and average trials per participant for each prior knowledge condition before excluding uniform readers. Tables S8, S9 and S10 illustrate pairwise comparisons for the probability to read between groups and for knowledge gains both between reading decisions and groups before excluding uniform readers.

Table S7

Number of Participants, Trials, and Average Trials per Participant by Group and Prior Knowledge for Pilot Experiments (Full Sample).

Experiment	Prior Knowledge	Experimental			Control		
		n(P)	n(T)	M(T/P)	n(P)	n(T)	M(T/P)
**Pilot Experiment 1**	0%	42	61	0.40	37	47	0.31
	33%	110	190	1.24	106	178	1.17
	67%	136	315	2.06	141	309	2.03
	100%	107	199	1.30	111	226	1.49
**Pilot Experiment 2**	0%	30	38	0.26	33	43	0.29
	33%	101	164	1.10	102	166	1.12
	67%	139	328	2.20	138	312	2.11
	100%	111	215	1.44	113	219	1.48

Note. n(P) = number of participants; n(C) = number of trials; M(C/P) = average trials per participant. Values are reported separately for each group (Experimental, Control) across prior knowledge conditions (0%, 33%, 67%, 100%).

Results from Analysis Containing All Participants (Main Experiments)

Figure 2 shows the plotted probability to read by group, score difference by reading behavior and score difference by group for both pilot experiments and both main experiments. Plots were calculated using the full sample before exclusion of uniform readers. Table S7 displays Number of Trials, number of participants and average trials per participant for each prior knowledge condition before excluding uniform readers. Tables S8, S9 and S10 illustrate pairwise comparisons for the probability to read

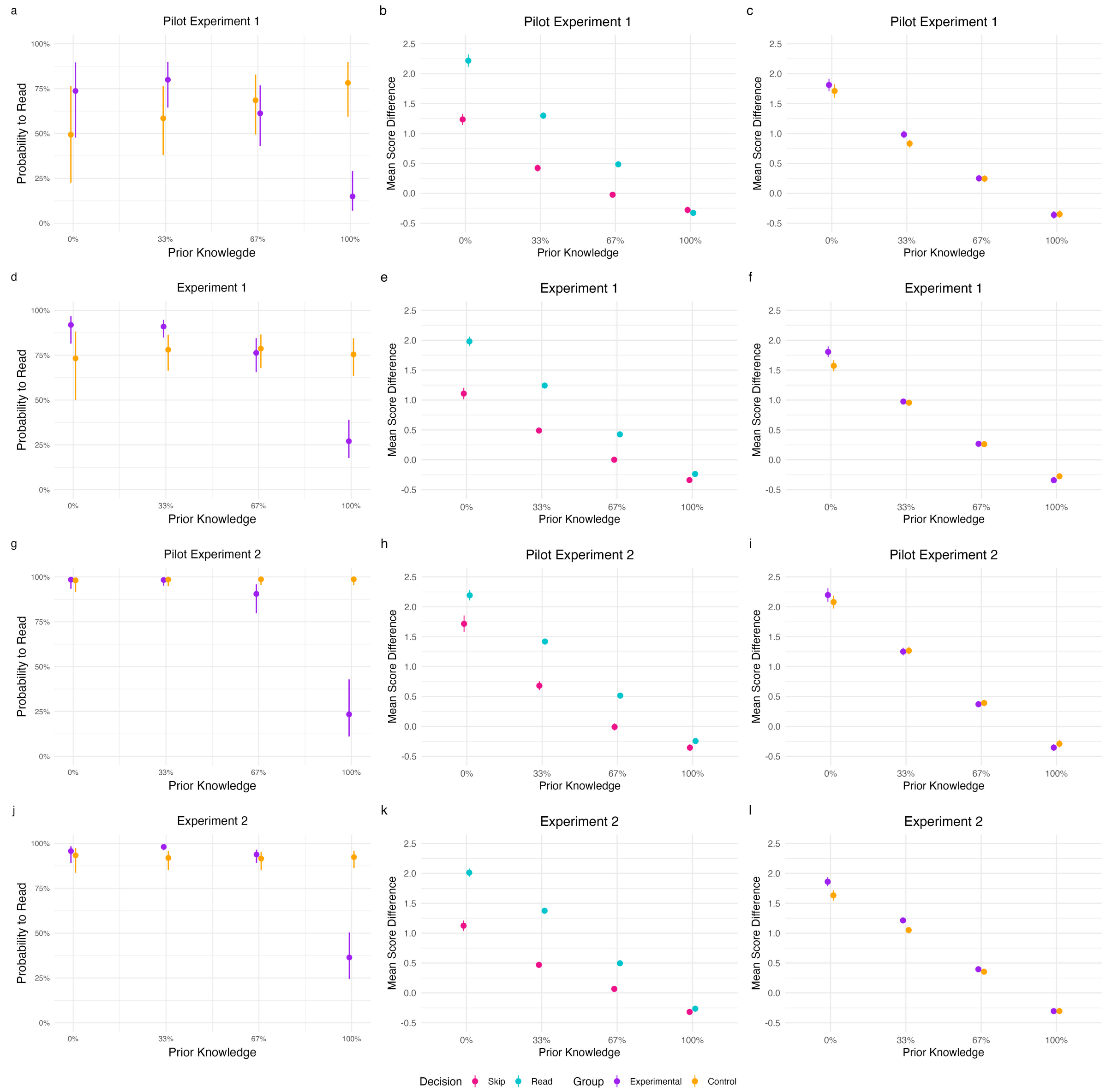

Figure 2

Probability to read by group, knowledge gains by reading decision and knowledge gains by group

between groups and for knowledge gains both between reading decisions and groups before excluding uniform readers.

Instruction and Stimulus Material

Instructions Before Pretest

"Thank you for taking part in this study! Next, you will take a knowledge quiz to assess your current knowledge on the topic around elephants in Botswana and Namibia. The

Table S8

Pairwise Contrasts for the Probability to Read Between Groups in Pilot Experiments (Full Sample).

**Prior Knowledge**	**Pilot Experiment 1**				**Pilot Experiment 2**			
	**OR**	**SE**	***z***	***p***	**OR**	**SE**	***z***	***p***
0%	0.35	0.29	-1.62	.207	0.38	0.72	-0.51	.610
33%	0.35	0.20	-1.80	.072	1.24	1.18	0.22	.824
67%	1.38	0.76	0.58	.565	0.87	0.56	-0.22	.824
100%	20.50	13.10	4.71	<.001***	0.13	0.08	-3.18	.002**

Note. Odds Ratios (*OR*) reflect the contrast: Experimental / Control for each prior knowledge condition. Values are based on estimated marginal means from hierarchical logistic regression models. *z*-values and *p*-values correspond to model-based comparisons. Significance levels: *$p < .05$, **$p < .01$, ***$p < .001$.

Table S9

Pairwise Contrasts for Score Differences by the Decision to Read in Pilot Experiments (Full Sample).

**Prior Knowledge**	**Pilot Experiment 1**				**Pilot Experiment 2**			
	**Diff**	**SE**	***t***	***p***	**Diff**	**SE**	***t***	***p***
0%	-0.98	0.14	-7.24	<.001***	-0.48	0.16	-2.98	<.003**
33%	-0.88	0.08	-11.44	<.001***	-0.74	0.08	-8.84	<.001***
67%	-0.51	0.06	-8.30	<.001***	-0.52	0.06	-8.11	<.001***
100%	0.05	0.07	0.67	.501	-0.11	0.07	-1.59	.113

Note. Values reflect estimated marginal mean differences in pretest-to-posttest scores between different decisions–reading or skipping– for each prior knowledge condition. Negative values indicate larger score gains in the "read" group. Significance levels: *$p < .05$, **$p < .01$, ***$p < .001$.

Table S10

Pairwise Contrasts for Score Differences Between Groups in Pilot Experiments (Full Sample).

**Prior Knowledge**	**Pilot Experiment 1**				**Pilot Experiment 2**			
	**Diff**	**SE**	***t***	***p***	**Diff**	**SE**	***t***	***p***
0%	0.10	0.15	0.66	.509	0.12	0.16	0.75	.451
33%	0.15	0.09	1.64	.101	-0.01	0.09	-0.17	.869
67%	0.01	0.08	0.07	.946	-0.02	0.08	-0.27	.785
100%	-0.01	0.09	-0.14	.891	-0.06	0.08	-0.77	.444

Note. Values reflect estimated marginal mean differences in pretest-to-posttest scores between experimental and control groups for each prior knowledge condition. Positive values indicate larger score gains in the experimental group. Significance levels: *$p < .05$, **$p < .01$, ***$p < .001$.

Table S11

Number of Participants, Trials, and Average Trials per Participant by Group and Prior Knowledge for Main Experiments (Full Sample).

Experiment	Prior Knowledge	Experimental			Control		
		n(P)	n(T)	M(T/P)	n(P)	n(T)	M(T/P)
**Experiment 1**	0%	49	55	0.22	39	56	0.22
	33%	166	289	1.14	163	272	1.10
	67%	237	601	2.38	228	598	2.41
	100%	221	573	2.26	223	562	2.27
**Experiment 2**	0%	55	82	0.32	42	60	0.24
	33%	162	279	1.09	152	259	1.02
	67%	238	582	2.27	234	596	2.34
	100%	220	593	2.32	232	615	2.41

Note. n(P) = number of participants; n(C) = number of trials; M(C/P) = average trials per participant. Values are reported separately for each group (Experimental, Control) across prior knowledge conditions (0%, 33%, 67%, 100%).

Table S12

Pairwise Contrasts for the Probability to Read Between Groups in Main Experiments (Full Sample).

Prior Knowledge	Experiment 1				Experiment 2			
	OR	SE	z	p	OR	SE	z	p
0%	4.14	2.88	2.04	.041*	1.59	1.10	0.68	.500
33%	2.83	1.16	2.55	.011*	4.53	2.04	3.56	<.001***
67%	0.87	0.33	-0.36	.721	1.40	0.58	0.81	.419
100%	0.12	0.05	-5.23	<.001***	0.05	0.02	-6.92	<.001***

Note. Odds Ratios (*OR*) reflect the contrast: Experimental / Control for each prior knowledge condition. Values are based on estimated marginal means from hierarchical logistic regression models. z-values and p-values correspond to model-based comparisons. Significance levels: *$p < .05$, **$p < .01$, ***$p < .001$.

quiz is comprised of 18 questions. Please answer each question to the best of your ability. You will not receive feedback on your answers at this time. When you are ready, you can begin with the questions."

Instructions After Pretest and Before Decisions to Read

"Next, you will read an article that discusses various aspects of elephant conservation. The article is divided into sections each covering a specific aspect of the topic. After each section of the article, you will be asked whether you want to continue reading the next section or skip it. Finally, you will take the same quiz as before once again to

Table S13

Pairwise Contrasts for Score Differences by the Decision to Read in Main Experiments (Full Sample).

Prior Knowledge	Pilot Experiment 1				Pilot Experiment 2			
	Diff	SE	*t*	*p*	Diff	SE	*t*	*p*
0%	-0.87	0.13	-7.01	<.001***	-0.89	0.11	-8.31	<.001***
33%	-0.75	0.06	-13.28	<.001***	-0.91	0.06	-15.02	<.001***
67%	-0.43	0.04	-10.26	<.001***	-0.43	0.04	-9.75	<.001***
100%	-0.10	0.04	-2.47	.014*	-0.05	0.04	-1.39	.166

Note. Values reflect estimated marginal mean differences in pretest-to-posttest scores between different decisions–reading or skipping– for each prior knowledge condition. Negative values indicate larger score gains in the "read" group. Significance levels: $^{*}p < .05$, $^{**}p < .01$, $^{***}p < .001$.

Table S14

Pairwise Contrasts for Score Differences Between Groups in Main Experiments (Full Sample).

Prior Knowledge	Pilot Experiment 1				Pilot Experiment 2			
	Diff	SE	*t*	*p*	Diff	SE	*t*	*p*
0%	0.23	0.13	1.79	.074	0.23	0.12	1.96	.050
33%	0.02	0.07	0.30	.768	0.16	0.07	2.44	.015*
67%	0.01	0.05	0.13	.900	0.04	0.52	0.79	.432
100%	-0.07	-0.07	-1.26	.201	-0.001	0.05	-0.02	.984

Note. Values reflect estimated marginal mean differences in pretest-to-posttest scores between experimental and control groups for each prior knowledge condition. Positive values indicate larger score gains in the experimental group. Significance levels: $^{*}p < .05$, $^{**}p < .01$, $^{***}p < .001$.

measure changes in your knowledge after reading the article" [1].

Instructions for Reading Decisions

Control Group: “In the following section, you can read about [chapter title]. Do you want to start reading?” Possible answers: “Yes, I want to read the next section of the article.” / ”No, I want to skip this section.”

Experimental Group: “Your knowledge about [chapter title] is at [0% / 33% / 67% / 100%] right now. By reading the following section of the article, you have the opportunity to learn more about the topic. Do you want to start reading?” Possible

[1] This last sentence was only included in the second pilot experiment and Experiment 2.

answers: “Yes, I want to read the next section of the article.” / ”No, I want to skip this section.”

Reading Material

Chapter 1: Botswana’s Elephant Population and Conservation Challenges

Botswana is home to one of the largest populations of African elephants, estimated at around 130,000, which constitutes roughly one-third of the entire elephant population in Africa. This conservation success is largely due to the country’s strong anti-poaching measures, expansive protected areas like Chobe National Park and the Okavango Delta, and an overall commitment to preserving wildlife. Botswana’s elephants are a significant draw for tourism, contributing to the nation’s economy, especially through eco-tourism. However, the increasing number of elephants has posed significant challenges, particularly in relation to human-elephant conflict. As elephant populations grow, the competition for land and resources intensifies, particularly in rural areas where humans and elephants live in close proximity. Elephants often stray into villages or farmlands in search of food, trampling crops, damaging infrastructure, and even posing a direct threat to human life. Farmers, who rely on their crops for their livelihoods, frequently face financial losses, leading to escalating tensions between local communities and wildlife conservationists. This conflict has sparked ongoing debates about the best ways to manage Botswana’s elephant population. On the one hand, the growing number of elephants is a conservation victory, but on the other, it has led to significant economic and social challenges for rural communities. Balancing the needs of wildlife and human populations is a delicate issue, with some proposing controversial solutions like trophy hunting or translocation as potential ways to manage elephant populations and reduce conflict.

Chapter 2: Historical Decline and Recovery of Elephant Populations

Historically, the African elephant population has faced severe declines. In the early 20th century, Africa was home to between 3 and 5 million elephants. However, poaching for ivory and habitat loss due to human encroachment caused a drastic reduction in their numbers. By the late 1970s, it was estimated that only about 1.3 million elephants

remained across the continent. The demand for ivory, particularly from international markets in Asia, led to widespread poaching, which decimated elephant populations. The global response to this crisis came in the form of the Convention on International Trade in Endangered Species of Wild Fauna and Flora (CITES), which implemented a ban on the international ivory trade in 1989. This measure significantly reduced poaching in some regions, allowing elephant populations to begin recovering, particularly in Southern Africa. Countries like Botswana, Namibia, Zimbabwe, and South Africa became strongholds for elephant conservation due to their robust anti-poaching efforts and the creation of large, protected areas. Despite these successes in Southern Africa, elephant populations in Central and West Africa remain critically endangered. In these regions, poaching continues at alarming rates, and habitat loss further threatens elephant survival. Conservationists argue that while the global ivory trade ban was an essential step, more comprehensive efforts are needed to protect elephants in all regions, not just in those countries with the resources and political will to enforce stringent conservation policies.

Chapter 3: The Growing Problem of Human-Elephant Conflict

[2]

As Botswana's elephant population has recovered and grown, the problem of human-elephant conflict has intensified. Elephants are large animals with significant needs for food and water. In times of drought or when natural food sources are scarce, elephants often wander into human settlements, destroying crops and water systems in their search for sustenance. Elephants, known for their intelligence and memory, may repeatedly return to areas where they have found food, exacerbating the conflict with local communities. The economic consequences of these encounters can be devastating for farmers, many of whom rely on subsistence agriculture to feed their families. Crop-raiding by elephants can result in the destruction of entire fields, leaving farmers with no harvest and, in some cases, no income for the season. Beyond economic damage, elephants can also pose a physical threat to people. While elephants are

[2] This chapter was excluded from analysis in the pilot experiments due to a technical issue.

generally peaceful, they can become aggressive if they feel threatened or provoked, leading to dangerous situations for those living near elephant territories. Climate change has further compounded the problem. As droughts become more frequent and severe, the availability of natural water and food sources declines, forcing elephants to roam further afield in search of resources. This increased movement brings elephants into more frequent contact with human populations, raising the risk of conflict. Conservationists and local governments have been working to develop strategies to reduce human-elephant conflict, but the problem remains a significant challenge for both wildlife conservation and community safety.

Chapter 4: Policy Approaches to Managing Elephant Populations

Various policy approaches have been explored to address the challenges posed by growing elephant populations, particularly in Botswana and neighboring countries. One of the most controversial methods has been culling, which involves the deliberate killing of elephants to reduce their numbers. This practice was widely used in South Africa's Kruger National Park during the mid-20th century, where thousands of elephants were culled to manage overpopulation and prevent environmental degradation caused by large herds. However, culling is a highly controversial method. It has faced widespread condemnation from animal rights groups and the international community, as it is seen as a cruel and inhumane solution. Additionally, research suggests that culling can disrupt elephant social structures, as it often involves killing entire family groups, which can traumatize surviving elephants, especially younger individuals who rely on older members of the herd for guidance. In Botswana, the government banned trophy hunting in 2014, but after a five-year hiatus, it was reintroduced in 2019. Trophy hunting allows wealthy tourists to pay for the opportunity to hunt elephants, with the revenue from these hunts purportedly going towards conservation efforts and local communities. Proponents argue that this is a way to manage elephant populations while generating much-needed funds for conservation. Critics, however, argue that the revenue benefits private operators more than local communities and that hunting disrupts elephant social dynamics, particularly when older males, who play a crucial role in herd

leadership, are killed. Other strategies, such as translocation—moving elephants from areas of high conflict to less populated regions—have also been employed, but these efforts are costly and logistically challenging. As Botswana continues to grapple with the challenge of managing its elephant population, finding a solution that balances conservation goals with the needs of local communities remains an ongoing struggle.

Chapter 5: Ethical and Economic Debates on Trophy Hunting and Trade

The debate over trophy hunting as a conservation tool is one of the most contentious issues in elephant management. Proponents of trophy hunting argue that, if properly regulated, it can serve as a valuable conservation tool. They claim that the fees collected from hunters can be used to fund conservation efforts and provide financial benefits to local communities. In areas like Botswana, where human-elephant conflict is prevalent, trophy hunting is seen as a way to reduce elephant numbers while also generating revenue. However, critics argue that the benefits of trophy hunting are overstated. They point out that much of the revenue from trophy hunting often goes to private hunting operators rather than directly benefiting conservation or local communities. Additionally, there are concerns that removing key individuals from elephant herds, particularly older males with large tusks, can have negative ecological consequences. These older males play an essential role in herd leadership, and their removal can increase aggression among younger males, leading to more frequent conflicts with humans. The ethical concerns surrounding trophy hunting are also significant. Many argue that killing elephants for sport is morally indefensible, particularly given their endangered status in many parts of Africa. Conservationists are increasingly calling for alternative strategies that focus on protecting elephants and their habitats without resorting to hunting. These alternatives include eco-tourism, which allows people to experience wildlife without harming it, and more sustainable forms of land management that can reduce human-elephant conflict.

Chapter 6: Innovative Approaches and the Future of Elephant Conservation

As traditional methods of managing elephant populations face increasing criticism, conservationists are turning to more innovative approaches to reduce human-elephant conflict and ensure the long-term survival of these majestic animals. One promising strategy is the creation of wildlife corridors, which are designated routes that allow elephants and other wildlife to move freely between protected areas. These corridors reconnect fragmented habitats, enabling elephants to migrate without entering human settlements, thereby reducing the likelihood of conflict. Another innovative solution is the use of bee fences. Elephants have a natural aversion to bees, and farmers in several African countries have begun using beehive fences to protect their crops. The sound and presence of bees are enough to deter elephants from raiding fields, and this approach has proven to be a cost-effective and environmentally friendly solution. Moreover, the honey produced from these beehives provides farmers with an additional source of income, creating a win-win situation for both conservation and local communities. Looking to the future, conservationists are focused on finding more sustainable, community based solutions to elephant conservation. Efforts to integrate local communities into conservation strategies are critical, as they are the ones most affected by human-elephant conflict. By involving local populations in decision-making processes and providing them with the tools and resources they need to coexist with elephants, conservationists hope to reduce conflict and ensure the continued survival of Africa's elephants.

Knowledge Test

correct answers are highlighted.

Chapter 1: Botswana's Elephant Population and Conservation Challenges

1. What percentage of Africa's elephant population is found in Botswana?

   a) 50%

   b) 30%

c) 40%

d) 25%

2. Why are farmers in Botswana facing economic challenges related to elephants?

a) Elephants block roadways frequently

b) Elephants raid crops and damage property

c) Tourism has reduced due to elephant overpopulation

d) Elephants are being overhunted

3. What is one reason Botswana has such a large elephant population?

a) Reintroduction of elephants from zoos

b) Import of elephants from neighboring countries

c) Lack of natural predators

d) Effective anti-poaching laws and large protected areas

Chapter 2: Historical Decline and Recovery of Elephant Populations

4. What was the estimated African elephant population at the beginning of the 20th century?

a) 1 million

b) 3 to 5 million

c) 10 million

d) 500,000

5. Why did the African elephant population decline significantly in the 20th century?

a) Elephants stopped reproducing

b) Increased poaching for ivory and habitat loss

c) Climate change reduced food sources

d) Disease wiped out large herds

6. Which global action helped reduce poaching by banning the ivory trade in 1989?

a) Paris Agreement

b) Geneva Convention

c) CITES

d) Kyoto Protocol

Chapter 3: The Growing Problem of Human-Elephant Conflict

[3]

7. Why do elephants often enter human settlements?

a) To escape predators

b) To find food and water during dry seasons

c) To seek companionship with humans

d) To migrate south for mating season

8. How has climate change worsened human-elephant conflict?

a) It has increased the aggression of elephants

b) It has reduced natural water and food resources, forcing elephants into human areas

c) It has caused elephants to migrate out of protected areas

d) It has made elephants immune to poaching efforts

9. What is a common consequence of human-elephant conflict in rural areas?

[3] This chapter was excluded from analysis in the pilot experiments due to a technical issue.

a) Farmers retaliate by attacking elephants

b) Elephants stop migrating altogether

c) Farmers give up on crop farming

d) Government compensation programs solve the conflict

Chapter 4: Policy Approaches to Managing Elephant Populations

10. What is culling, and why has it been used in elephant population management?

a) Selective breeding to increase numbers

b) Selective killing of elephants to reduce population numbers

c) Capturing elephants for zoos

d) Genetic modification to control breeding

11. Why was trophy hunting reintroduced in Botswana in 2019?

a) To encourage sport hunting as a new industry

b) To increase tourism opportunities

c) To promote elephant migration to neighboring countries

d) To control elephant populations and generate revenue for conservation

12. What is one major criticism of trophy hunting in elephant conservation?

a) It increases poaching

b) It disrupts elephant social structures and primarily benefits private operators

c) It has no measurable effect on population control

d) It results in less revenue for local communities

Chapter 5: Ethical and Economic Debates on Trophy Hunting and Trade

13. What is a key argument made in favor of trophy hunting?

  a) It increases elephant populations

  b) It generates revenue that can be reinvested into conservation

  c) It encourages elephants to reproduce faster

  d) It completely prevents poaching

14. What is a common criticism of the economic benefits of trophy hunting?

  a) Most revenue goes to private companies instead of local communities

  b) It causes overpopulation in protected areas

  c) It lowers tourism opportunities in those areas

  d) Hunting licenses are too cheap to benefit conservation

15. How can trophy hunting negatively impact elephant herds?

  a) It results in the extinction of older elephants

  b) It leads to higher mortality rates among younger elephants

  c) It increases aggression in younger males when older males are removed

  d) It disrupts the elephants' migration routes

Chapter 6: Innovative Approaches and the Future of Elephant Conservation

16. What is the purpose of wildlife corridors in elephant conservation?

  a) To keep elephants confined to specific areas

  b) To track elephants with GPS systems

  c) To prevent elephants from migrating at all

d) To allow elephants to migrate between protected areas without entering human settlements

17. Why are bee fences considered effective in preventing elephants from raiding crops?

    a) Elephants are afraid of the sound of bees and avoid them

    b) Bees can physically harm elephants

    c) Bees create barriers that elephants cannot cross

    d) Bees communicate with elephants to stay away

18. How do wildlife corridors help reduce human-elephant conflict?

    a) They increase land available for human settlements

    b) They restrict elephants from accessing water and food

    c) They provide alternative migration routes for elephants, away from human-inhabited areas

    d) They keep elephants from moving between protected areas
